# Hypervalent iodine(III)-induced methylene acetoxylation of 3-oxo-N-substituted butanamides

**DOI:** 10.3762/bjoc.7.167

**Published:** 2011-10-19

**Authors:** Wei-Bing Liu, Cui Chen, Qing Zhang, Zhi-Bo Zhu

**Affiliations:** 1 School of Chemistry and Life Science, Guangdong University of Petrochemical Technology, Maoming 525000, China; 2 College of Pharmaceutical Sciences, Southern Medical University, Guangzhou 510515, China

**Keywords:** 1-carbamoyl-2-oxopropyl acetate derivatives, C-hetero bond, (diacetoxyiodo)benzene, methylene acetoxylation

## Abstract

1-Carbamoyl-2-oxopropyl acetate derivatives were synthesized through an acetoxylation process to methylene with the aid of (diacetoxyiodo)benzene (DIB) as the oxidant. Not only mild reaction conditions, but also excellent yields and good substrate scope make the present protocol potentially useful in organic synthesis.

## Introduction

**Scheme 1:**
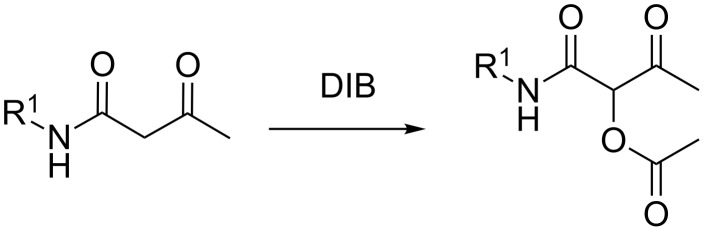
Synthesis of 1-carbamoyl-2-oxopropyl acetates.

Carbon–carbon, carbon–heteroatom bond formation leading to useful molecular structures is one of the most interesting and challenging research topics in organic chemistry [1–14]. Indeed, direct oxidative C–H bond functionalization provides an atom-economical and efficient pathway to achieve these goals. Representative examples have been elegantly utilized not only in academic research, but also in the production of a variety of fine chemicals, such as pharmaceuticals, agrochemicals, and intermediates [15–18]. The field of chemistry concerning organic polyvalent iodine compounds has witnessed a great expansion during the last few decades, an expansion which continues at an increasing pace [19–30]. The availability of iodine(III) and iodine(V) compounds and the development of new reagents, along with their low toxicity, ready availability, easy handling, clean transformation and reactivity, their selectivity under a variety of conditions, and their tolerance to different functional groups make these compounds valuable tools in organic synthesis [31–36]. Our interest in the chemistry of polyvalent iodine(III) reagents [37–39] prompted us to exploit the reactivity of (diacetoxyiodo)benzene (DIB). We report herein the use of DIB, as a nucleophile and oxidant, to perform an acetoxylation reaction with 3-oxo-N-substituted butanamides (Scheme 1).

## Results and Discussion

Initially, we employed 3-oxo-*N*-phenylbutanamide (**1a**) as the model substrate and tried to establish an effective reaction system for the synthesis. The results are shown in Table 1. It was found that the reaction afforded the desired product 1-(phenylcarbamoyl)-2-oxopropyl acetate (**2a**) by using DIB as the additive, and the optimum reaction time was 2 hours (Table 1, entries 1–3), whereas almost no desired product was obtained when Lewis acids were added (Table 1, entries 4–6). Among the various solvents examined, dioxane, DCE and DMF were practical solvents (Table 1, entries 2, 7–9). It is noteworthy that the reaction led to an obvious decrease of the yield of **2a** when either 0.5 or 2 equiv of DIB were used (Table 1, entries 11 and 13) compared to 1.3 equiv (Table 1, entry 12), which was found to be the optimum amount of DIB (Table 1, entries 11–13).

**Table 1:** Optimization of reaction conditions.^a^

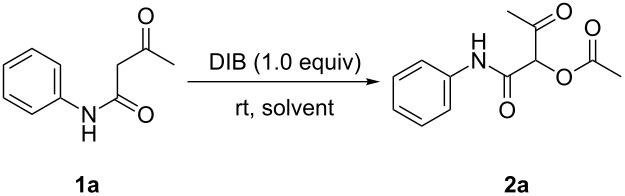				

entry	solvent	additive (1.0 equiv)	time (h)	yield (%)^b^

1	dioxane	—	1	66
2	dioxane	—	2	80
3	dioxane	—	3	81
4	dioxane	FeCl_3_	2	trace
5	dioxane	ZnCl_2_	2	trace
6	dioxane	CuCl_2_	2	trace
7	cyclohexane	—	2	36
8	DCE	—	2	82
9	DMF	—	2	71
10	DMSO	—	2	47
11^c^	DCE	—	2	35
12^d^	DCE	—	2	89
13^e^	DCE	—	2	75

^a^**1a** (0.25 mmol), solvent (2 mL), DIB (1.0 equiv); ^b^GC yield; ^c^DIB (0.5 equiv); ^d^DIB (1.3 equiv); ^e^DIB (2.0 equiv).

**Scheme 2:**
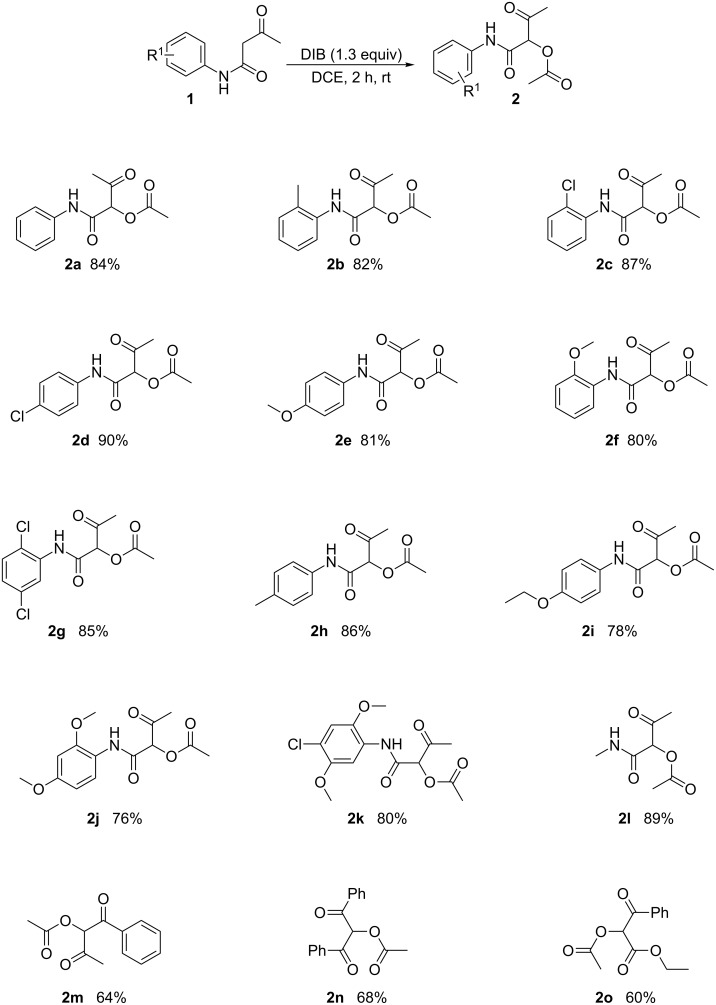
The synthesis of 1-carbamoyl-2-oxopropyl acetates. Conditions: **1** (1.0 mmol), DCE (2 mL), DIB (1.3 equiv); %: Isolated yield.

To explore the substrate scope and limitations of this reaction, a range of 3-oxo-*N*-phenylbutanamides were then examined under the optimized reaction conditions. The results are shown in Scheme 2.

We found that the reaction led to the corresponding products **2a–2l** in excellent isolated yields with all substrates. The reaction appears to be quite tolerant to differences in the position, number and electronic contribution of the substituent on the benzene ring. For example, the reactions of 3-oxo-*N*-phenylbutanamide, *N*-(4-methoxyphenyl)-3-oxobutanamide, *N*-(2-methoxyphenyl)-3-oxobutanamide, *N*-(2,5-dichlorophenyl)-3-oxobutanamide, *N*-(2,4-dimethoxyphenyl)-3-oxobutanamide as well as *N*-(4-chloro-2,5-dimethoxyphenyl)-3-oxobutanamide all lead to the corresponding products (**2a**, **2e**, **2f**, **2g**, **2j**, and **2k**, respectively) in excellent isolated yield. Similarly, the reactions of other N-(alkylsubstituted)-3-oxobutanamides were investigated, such as that of *N*-methyl-3-oxobutanamide (**1l**), which led to 1-(methylcarbamoyl)-2-oxopropyl acetate in 89% yield. Furthermore, we applied this method to non-carbamoyl 1,3-dicarbonyl compounds. These substrates, namely 1-phenylbutane-1,3-dione, 1,3-diphenylpropane-1,3-dione and ethyl 3-oxo-3-phenylpropanoate, all produced products in moderate isolated yields (**2m**, **2n**, **2o**).

**Scheme 3:**
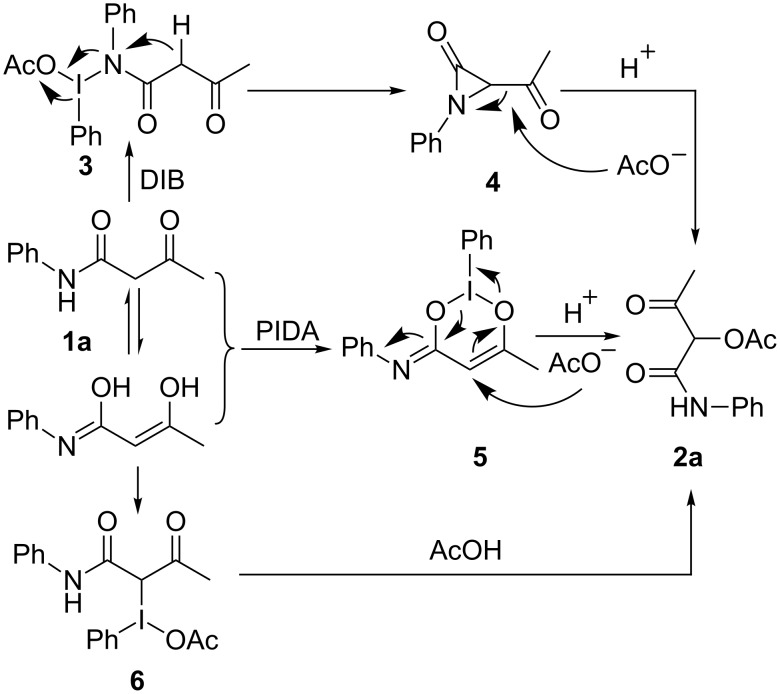
Possible reaction mechanism.

A plausible mechanism for the described transformation can be rationalized as shown in Scheme 3. The reaction initiates with the attack of the lone-pair electrons of the carbamoyl nitrogen [39–41] or carbonyl oxygen [42–45] on the iodine(III) of DIB, forming intermediates **3** and **5**, respectively. Alternatively, DIB attacks the C–C double bond of the enol derived from **1a** and forms intermediate **6** [46–47]. The subsequent N–I, O–I and C–I bond cleavage along with the nucleophilic attack of the acetate ion on the C–N or C–C double bond of the intermediate **4**, **5** or **6** affords the final product **2a**.

## Conclusion

In conclusion, we have shown an efficient and operationally simple method to synthesize 1-carbamoyl-2-oxopropyl acetate derivatives. The readily accessible starting materials, cheap oxidant DIB, as well as the mild reaction conditions and excellent yields make the present protocol potentially useful in organic synthesis. Further studies on the application to more valuable compounds and detailed investigations of the reaction mechanism are in progress.

## Supporting Information

File 1 Experimental details and copies of NMR spectra.
