# Supplementary material for: Hypervalent iodine(III)-induced methylene acetoxylation of 3-oxo-N-substituted butanamides
Source: Beilstein J Org Chem. 2011 Oct 19;7:1436–40. doi: 10.3762/bjoc.7.167 (PMC3205772; doi:10.3762/bjoc.7.167)
Supplement: File 1 — Experimental details and copies of NMR spectra. [file Beilstein_J_Org_Chem-07-1436-s001.pdf]

Supporting Information

for

Hypervalent iodine(III)-induced methylene acetoxylation of 3-oxo-N-substituted butanamides

Wei-Bing Liu[1], Cui Chen[1], Qing Zhang[1] and Zhi-Bo Zhu*[2]

Address: [1]School of Chemistry and Life Science, Guangdong University of Petrochemical Technology, Maoming 525000, China and

[2]College of Pharmaceutical Sciences, Southern Medical University, Guangzhou 510515, China

Email: Zhi-Bo Zhu - zzb24@yahoo.com.cn

* Corresponding author

Experimental details and copies of NMR spectra.

General methods: All the reactions were carried out at room temperature in a Schlenk tube equipped with a magnetic stirring bar. Solvents and all reagents were used as received. ^{1}H NMR spectra were recorded in $CDCl_3$ at 400 MHz and ^{13}C NMR spectra were recorded in $CDCl_3$ at 100 MHz. GC–MS was obtained by electron impact ionization (EI). TLC was performed with commercially prepared 100–400 mesh silica gel plates (GF_{254}), and visualized at 254 nm. All the other chemicals were purchased from Aldrich Chemicals.

Typical procedure for the synthesis of 1-(phenylcarbamoyl)-2-oxopropyl acetate (2a): To a 10 mL Schlenk tube was added DIB (419 mg, 1.3 mmol), DCE (2 mL) and 3-oxo-*N*-phenylbutanamide (**1a**) (177 mg, 1.0 mmol). The mixture was stirred at rt for 2 h. The solution was directly subjected to isolation by PTLC (GF254), eluted with a 10:4 petroleum ether/ethyl acetate mixture, which furnished **2a** (197.4 mg, 84%) as a colorless solid.

Characterization data for compounds:

1-(Phenylcarbamoyl)-2-oxopropyl acetate (2a) [1]

^{1}H NMR ($CDCl_3$, 400 MHz) δ 8.09 (s, 1H), 7.51–7.49 (d, *J* = 8 Hz, 2H), 7.33–7.30 (t, 2H), 7.15–7.12 (t, 1H), 5.61 (s, 1H), 2.44 (s, 3H), 2.27 (s, 3H); ^{13}C NMR ($CDCl_3$, 100 MHz) δ 200.2, 168.8, 161.2, 136.6, 129.3, 125.5, 120.5, 79.5, 28.0, 20.8; MS (EI) *m/z* (%): 193.10 (100.00), 235.05 (20.27).

1-(*o*-Tolylcarbamoyl)-2-oxopropyl acetate (2b)

Colorless solid; mp: 83–84 ºC; ^{1}H NMR ($CDCl_3$, 400 MHz) δ 8.07 (s, 1H), 7.74–7.72 (d, *J* = 8 Hz, 2H), 7.19–7.16 (m, 3H), 7.10–7.06 (m, 1H), 5.63 (s, 1H), 2.43 (s, 3H), 2.26 (s, 3H), 2.23 (s, 3H); ^{13}C NMR ($CDCl_3$, 100 MHz) δ 200.4, 168.8, 161.4, 134.5, 130.8, 129.8, 127.0, 126.1, 123.1, 79.4, 27.9, 20.7, 17.6; MS (EI) *m/z* (%): 207.05 (100.00), 248.65 (21.69); Anal. calcd for $C_{13}H_{15}NO_4$: C, 62.64; H, 6.07; found: C, 62.55; H, 6.25.

1-(2-Chlorophenylcarbamoyl)-2-oxopropyl acetate (2c)

Pale yellow oil; ^{1}H NMR ($CDCl_3$, 400 MHz) δ 8.74 (s, 1H), 8.29–8.27 (d, *J* = 8 Hz, 1H), 7.38–7.36 (d, *J* = 8 Hz, 1H), 7.29–7.26 (m, 1H), 7.09–7.05 (m, 1H), 5.66 (s, 1H), 2.46 (s, 3H), 2.29 (s, 3H); ^{13}C NMR ($CDCl_3$, 100 MHz) δ 199.3, 168.7, 161.4, 133.6, 129.5, 128.1, 125.8, 123.6, 121.7, 79.3, 28.0, 20.7; MS (EI) *m/z* (%): 184.95 (100.00), 268.95 (19.58); Anal. calcd for $C_{12}H_{12}ClNO_4$: C, 53.44; H, 4.49; found: C, 53.41; H, 4.57.

1-(4-Chlorophenylcarbamoyl)-2-oxopropyl acetate (2d)

Pale yellow oil; IR ν_{max} (KBr): 3319, 2927, 1933, 1687, 1597, 1533, 1460, 1371, 1091, 1020, 920, 825, 773, 686 cm^{-1}; ^{1}H NMR ($CDCl_3$, 400 MHz) δ 8.08 (s, 1H), 7.46–7.44 (d, *J* = 8 Hz, 2H), 7.29–7.27 (d, *J* = 8 Hz, 2H), 5.60 (s, 1H), 2.44 (s, 3H), 2.27 (s, 3H); ^{13}C NMR ($CDCl_3$, 100 MHz) δ 199.9, 168.5, 160.9, 134.9, 130.2, 129.1, 121.4, 79.0, 27.7, 20.5; MS (EI) *m/z* (%): 43.00 (100.00), 268.90 (11.73); Anal. calcd for $C_{12}H_{12}ClNO_4$: C, 53.44; H, 4.49; found: C, 53.55; H, 4.39.

1-(4-Methoxyphenylcarbamoyl)-2-oxopropyl acetate (2e)

Pale yellow solid; mp:80–82 ºC; IR ν_{max} (KBr): 3315, 2933, 1736, 1678, 1609, 1514, 1450, 1400, 1365, 1222, 1085, 1028, 912, 829, 707cm^{-1}; ^{1}H NMR ($CDCl_3$, 400 MHz) δ 7.98 (s, 1H), 7.39–7.37 (d, *J* = 8 Hz, 2H), 6.85–6.83 (d, *J* = 8 Hz, 2H), 5.60 (s, 1H), 3.76 (s, 3H), 2.44 (s, 3H), 2.26 (s, 3H); ^{13}C NMR ($CDCl_3$, 100 MHz) δ 199.9, 168.5, 160.8, 157.0, 129.3, 122.0, 114.2, 79.1, 55.4, 27.7, 20.5; MS (EI) *m/z* (%): 123.00 (100.00), 264.95 (40.65); Anal. calcd for $C_{13}H_{15}NO_5$: C, 58.86; H, 5.70; found: C, 58.71; H, 5.52.

1-(2-Methoxyphenylcarbamoyl)-2-oxopropyl acetate (2f)

Pale yellow oil; IR ν_{max} (KBr): 3408, 2941, 1751, 1687, 1602, 1533, 1111, 1029, 918, 752, 692 cm^{-1}; ^{1}H NMR ($CDCl_3$, 400 MHz) δ 8.70 (s, 1H), 8.25–8.23 (m, 1H), 6.94–6.92 (m, 1H), 6.90–6.84 (m, 2H), 5.62 (s, 1H), 3.86 (s, 3H), 2.43 (s, 3H), 2.26 (s, 3H); ^{13}C NMR ($CDCl_3$, 100 MHz) δ 199.4, 168.5, 160.7, 148.2, 126.1, 124.7, 120.9, 119.8, 110.0, 79.3, 55.7, 27.6, 20.4; MS (EI) *m/z* (%): 123.00 (100.00), 264.95 (22.81); Anal. calcd for $C_{13}H_{15}NO_5$: C, 58.86; H, 5.70; found: C, 58.90; H, 5.88.

1-(2,5-Dichlorophenylcarbamoyl)-2-oxopropyl acetate (2g)

White solid; mp: 75–77 ºC; IR ν_{max} (KBr):3433, 2924, 1707, 1583, 1525, 1458, 1406, 1280, 1211, 1089, 1045, 916, 873, 808cm^{-1}; ^{1}H NMR ($CDCl_3$, 400 MHz) δ 8.45 (s, 1H), 7.23 (s, 1H), 7.00–6.99 (d, *J* = 4 Hz, 1H), 6.98–6.97 (d, *J* = 4 Hz, 1H), 5.64 (s, 1H), 2.45 (s, 3H), 2.29 (s, 3H); ^{13}C NMR ($CDCl_3$, 100 MHz) δ 199.8, 168.3, 160.8, 134.8, 129.8, 129.5, 125.4, 124.7, 121.2, 78.9, 27.2, 20.4; MS (EI) *m/z* (%): 43.00 (100.00), 302.85 (6.82); Anal. calcd for $C_{12}H_{11}Cl_2NO_4$: C, 47.39; H, 3.65; found: C, 47.55; H, 3.60.

1-(*p*-Tolylcarbamoyl)-2-oxopropyl acetate (2h)

Orange oil; mp: 88–89 ºC; IR ν_{max} (KBr): 3365, 1733, 1604, 1597, 1411, 1228, 1089, 916, 819, 727cm^{-1}; ^{1}H NMR ($CDCl_3$, 400 MHz) δ 8.20 (s, 1H), 7.35–7.33 (d, *J* = 8 Hz, 2H), 6.08–7.06 (d, *J* = 8 Hz, 2H), 5.57 (s, 1H), 2.39 (s, 3H), 2.26 (s, 3H), 2.21 (s, 3H); ^{13}C NMR ($CDCl_3$, 100 MHz) δ 200.0, 168.7, 161.1, 134.9, 133.9, 129.6, 120.3, 79.2, 27.6, 20.8, 20.4; MS (EI) *m/z* (%): 107.05 (100.00), 248.95 (22.08); Anal. calcd for $C_{13}H_{15}NO_4$: C, 62.64; H, 6.07; found: C, 62.49; H, 5.88.

1-(4-Ethoxyphenylcarbamoyl)-2-oxopropyl acetate (2i)

Orange solid; mp: 92–93 ºC; IR ν_{max} (KBr): 3356, 2926, 1736, 1678, 1516, 1450, 1365, 1240, 1043, 920, 833, 711cm^{-1}; ^{1}H NMR ($CDCl_3$, 400 MHz) δ 7.97 (s, 1H), 7.38–7.36 (d, *J* = 8 Hz, 2H), 6.84–6.82 (d, *J* = 8 Hz, 2H), 5.60 (s, 1H), 4.00–3.95 (q, *J* = 6.4 Hz, 2H), 2.43 (s, 3H), 2.26 (s, 3H), 1.39–1.35 (t, *J* = 6.4 Hz, 3H); ^{13}C NMR ($CDCl_3$, 100 MHz) δ 199.8, 168.5, 160.7, 156.4, 129.2, 122.0, 114.7, 79.2, 63.6, 27.7, 20.5, 14.7; MS (EI) *m/z* (%): 137.05 (100.00), 278.95 (39.33); Anal. calcd for $C_{14}H_{17}NO_5$: C, 60.21; H, 6.14; found: C, 60.33; H, 6.11

1-(2,4-Dimethoxyphenylcarbamoyl)-2-oxopropyl acetate (2j)

Orange solid; mp: 85–86 ºC; IR ν_{max} (KBr): 3408, 2951, 2360, 1732, 1689, 1610, 1537, 1456, 1400, 1365, 1213, 1033, 920, 827, 686 cm^{-1}; ^{1}H NMR ($CDCl_3$, 400 MHz) δ 8.44 (s, 1H), 8.08–8.06 (d, *J* = 8 Hz, 1H), 6.42–6.37 (m, 2H), 5.58 (s, 1H), 3.80 (s, 3H), 3.72 (s, 3H), 2.39 (s, 3H), 2.22 (s, 3H); ^{13}C NMR ($CDCl_3$, 100 MHz) δ 199.4, 168.5, 160.3, 157.0, 149.6, 120.7, 119.7, 103.6, 98.5, 79.2, 55.7, 55.4, 27.6, 20.4; MS (EI) *m/z* (%): 153.00 (100.00), 294.95 (31.26); Anal. calcd for $C_{14}H_{17}NO_6$; C, 56.94; H, 5.80; found: C, 57.02; H, 5.88

1-(4-Chloro-2,5-dimethoxyphenylcarbamoyl)-2-oxopropyl acetate (2k)

Orange solid; mp: 101–102 ºC; IR ν_{max} (KBr): 3394, 2945, 1732, 1687, 1600, 1533, 1450, 1400, 1213, 727, 694 cm^{-1}; ^{1}H NMR ($CDCl_3$, 400 MHz) δ 8.71 (s, 1H), 8.10 (s, 1H), 6.89 (s, 1H), 5.61 (s, 1H), 3.85 (s, 3H), 3.84 (s, 3H), 2.44 (s, 3H), 2.28 (s, 3H); ^{13}C NMR ($CDCl_3$, 100 MHz) δ 199.5, 168.5, 160.8, 149.0, 142.2, 125.5, 116.9, 112.4, 104.9, 79.1, 56.7, 56.5, 27.7, 20.4; MS (EI) *m/z* (%): 186.90 (100.00), 328.90 (45.95); Anal. calcd for $C_{14}H_{16}ClNO_6$: C, 51.00; H, 4.89; found: C, 51.13; H, 4.91.

1-(Methylcarbamoyl)-2-oxopropyl acetate(2l)

Pale yellow oil; ^{1}H NMR ($CDCl_3$, 400 MHz) δ 6.60 (s, 1H), 5.42 (s, 1H), 2.76–2.75 (d, *J* = 4 Hz, 3H), 2.33 (s, 3H), 2.13 (s, 3H); ^{13}C NMR ($CDCl_3$, 100 MHz) δ 199.9, 168.8, 163.8, 79.1, 27.8, 26.2, 20.5; MS (EI) *m/z* (%): 89.05 (100.00), 173.15 (1.05); Anal. calcd for $C_7H_{11}NO_4$: C, 48.55; H, 6.40; found: C, 48.31; H, 6.57.

1,3-Dioxo-1-phenylbutan-2-yl acetate (2m) [2,3]

IR ν_{max} (KBr): 3446, 1718, 1627, 1394, 1224, 1112, 1018, 918, 696 cm^{-1}; ^{1}H NMR ($CDCl_3$, 400 MHz) δ 7.99–7.97 (d, *J* = 8 Hz, 2H), 7.61–7.58 (m, 1H), 7.51–7.47 (m, 2H), 6.24 (s, 1H), 2.27 (s, 3H), 2.21 (s, 3H); ^{13}C NMR ($CDCl_3$, 100 MHz) δ 199.7, 191.1, 169.6, 134.5, 130.4, 129.7, 129.0, 82.5, 27.1, 20.8; MS (EI) *m/z* (%): 105.00 (100.00).

1,3-Dioxo-1,3-diphenylpropan-2-yl acetate (2n) [3]

IR ν_{max} (KBr): 3646, 2943, 1720, 1690, 1600, 1444, 1375, 1199, 1004, 906, 713 cm^{-1}; ^{1}H NMR ($CDCl_3$, 400 MHz) δ 8.02–8.00 (d, *J* = 8 Hz, 4H), 7.60–7.57 (m, 2H), 7.47–7.40 (m, 4H), 6.98 (s, 1H), 2.21 (s, 3H); ^{13}C NMR ($CDCl_3$, 100 MHz) δ 191.2, 169.5, 134.4, 129.6, 129.0, 127.9, 80.1, 67.3, 20.8; MS (EI) *m/z* (%): 105.05 (100.00), 282.00 (0.77).

Ethyl 2-acetoxy-3-oxo-3-phenylpropanoate (2o) [3,4]

IR ν_{max} (KBr): 3429, 2983, 1747, 1691, 1593, 1450, 1379, 1238, 1103, 921, 717, 684 cm^{-1}; ^{1}H NMR ($CDCl_3$, 400 MHz) δ 7.94–7.92 (d, *J* = 8 Hz, 2H), 7.56–7.53 (t, 1H), 7.44–7.40 (m, 2H), 6.27 (s, 1H), 4.19–4.14 (q, 2H), 2.14 (s, 3H), 1.15–1.11 (t, 3H); ^{13}C NMR ($CDCl_3$, 100 MHz) δ 189.7, 169.6, 165.2, 134.3, 134.2, 129.2, 128.8, 74.5, 62.5, 20.5, 13.9; MS (EI) *m/z* (%):105.05 (100.00), 249.95 (0.25).

NMR spectra

2a

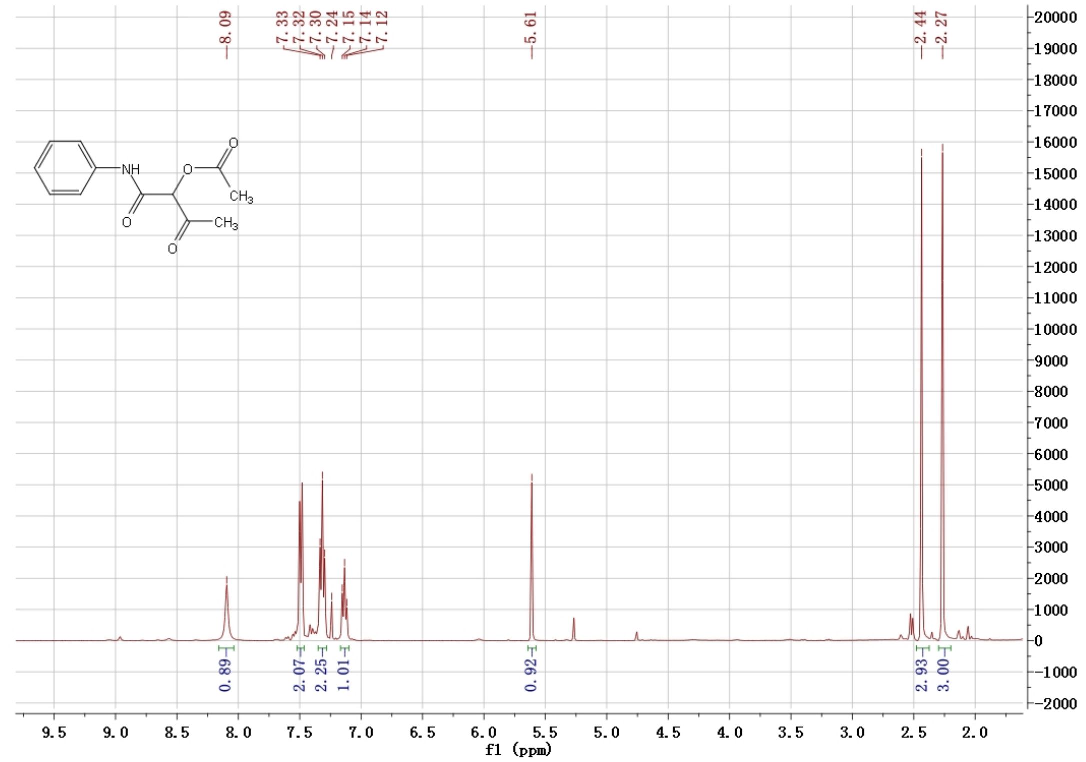

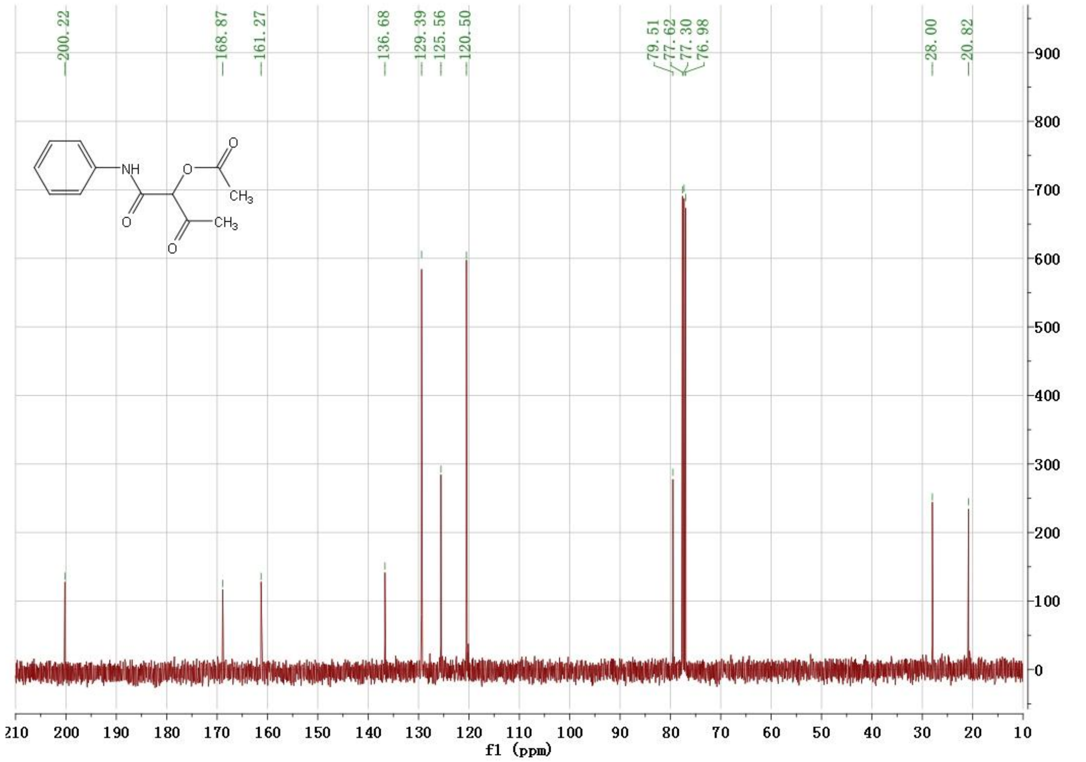

2b

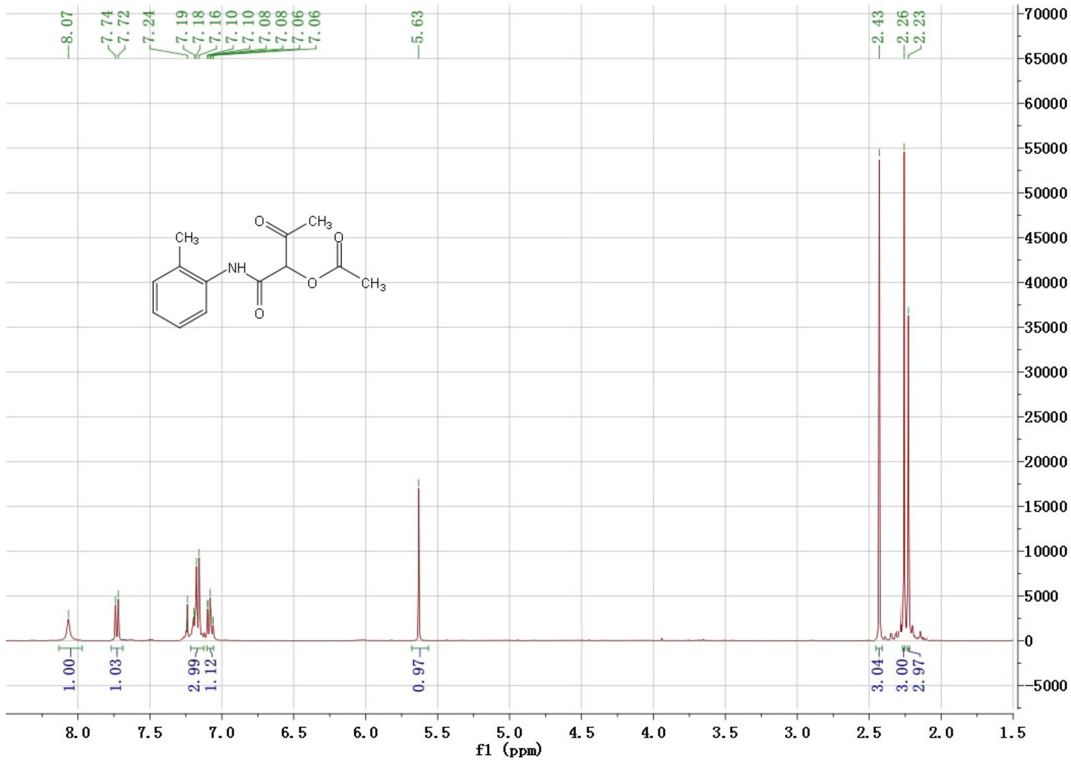
8.07
7.74
7.72
7.24
7.19
7.18
7.16
7.10
7.10
7.08
7.06
7.06
5.63
2.43
2.26
2.23
CH3
NH
O
O
CH3
O
O
CH3
1.00
1.03
2.99
1.12
0.97
3.04
3.00
2.97
f1 (ppm)

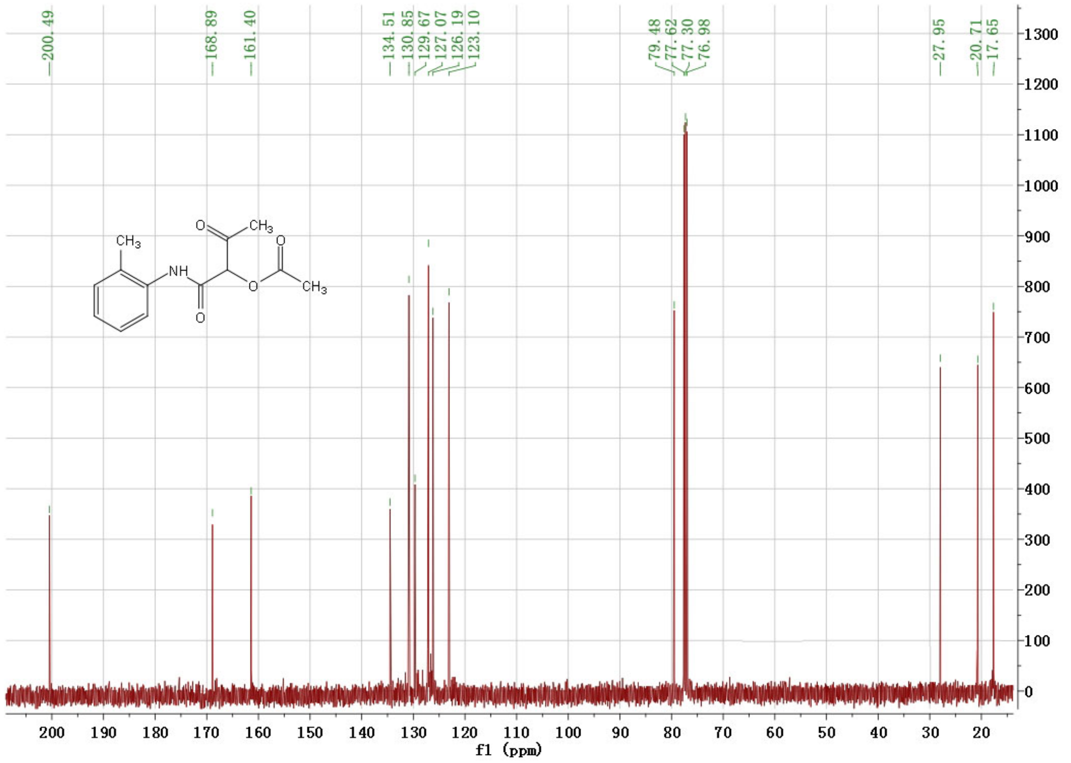
200.49
168.89
161.40
134.51
130.85
129.67
127.07
126.19
123.10
79.48
77.62
77.30
76.98
27.95
20.71
17.65
CH3
NH
O
O
CH3
O
O
CH3
f1 (ppm)

2c

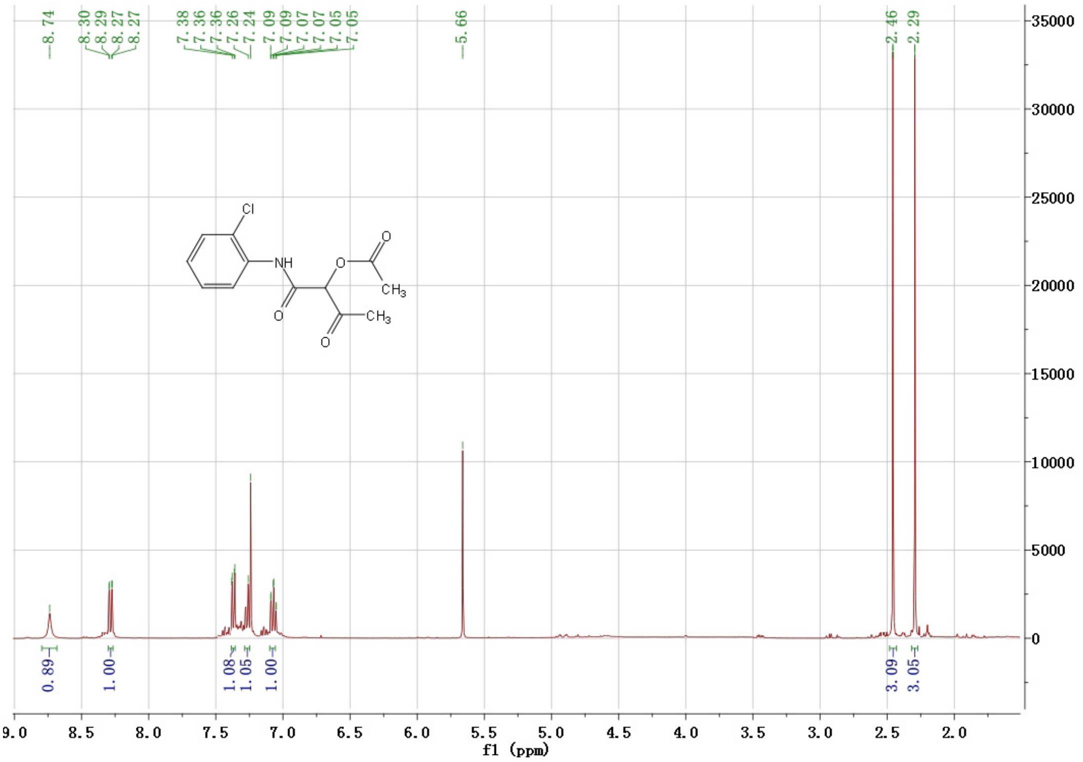

8.74
8.30
8.29
8.27
8.27
7.38
7.36
7.36
7.26
7.24
7.09
7.09
7.07
7.07
7.05
7.05
5.66
2.46
2.29
Cl
NH
O
O
CH3
O
CH3
O
0.89
1.00
1.08
1.05
1.00
3.09
3.05
f1 (ppm)

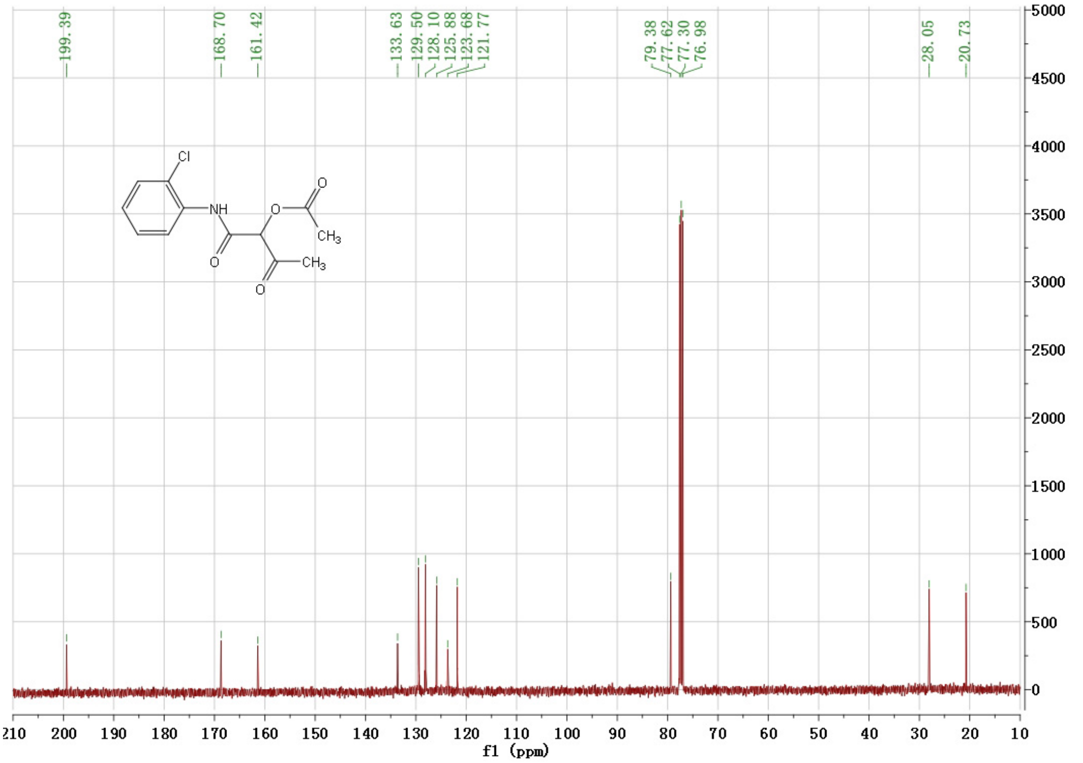

199.39
168.70
161.42
133.63
129.50
128.10
125.88
123.68
121.77
79.38
77.62
77.30
76.98
28.05
20.73
Cl
NH
O
O
CH3
O
CH3
O
f1 (ppm)

2d

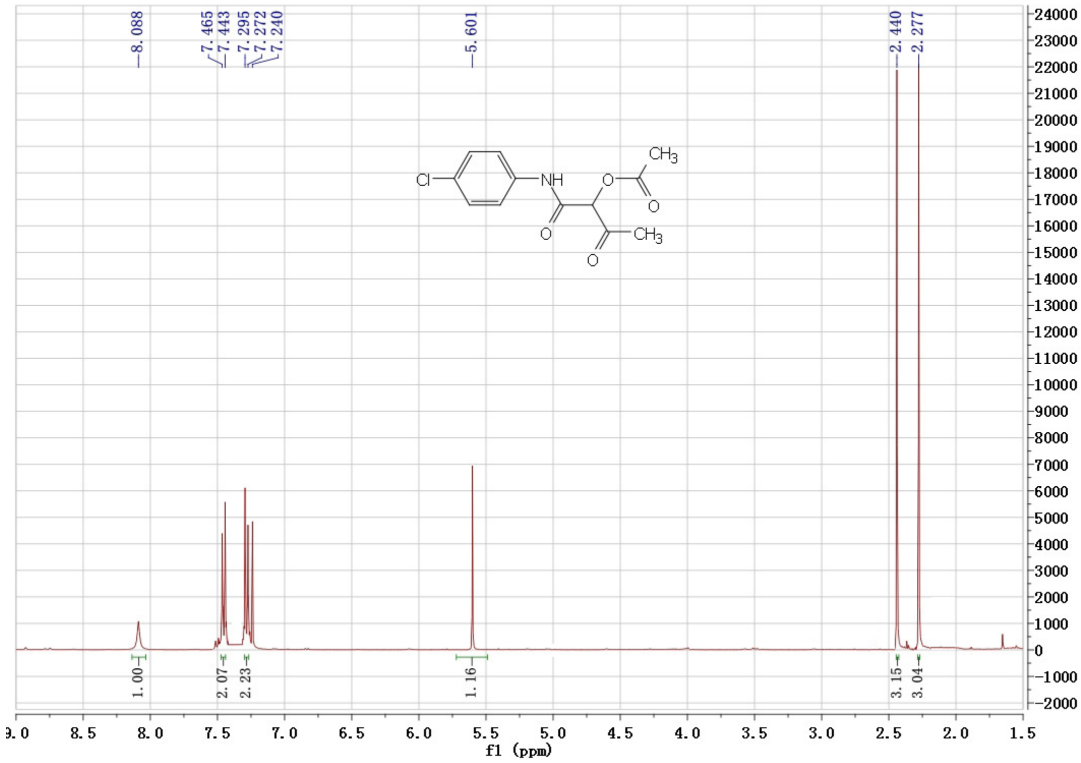
8.088
7.465
7.443
7.295
7.272
7.240
5.601
2.440
2.277
Cl
NH
O
CH3
1.00
2.07
2.23
1.16
3.15
3.04
f1 (ppm)

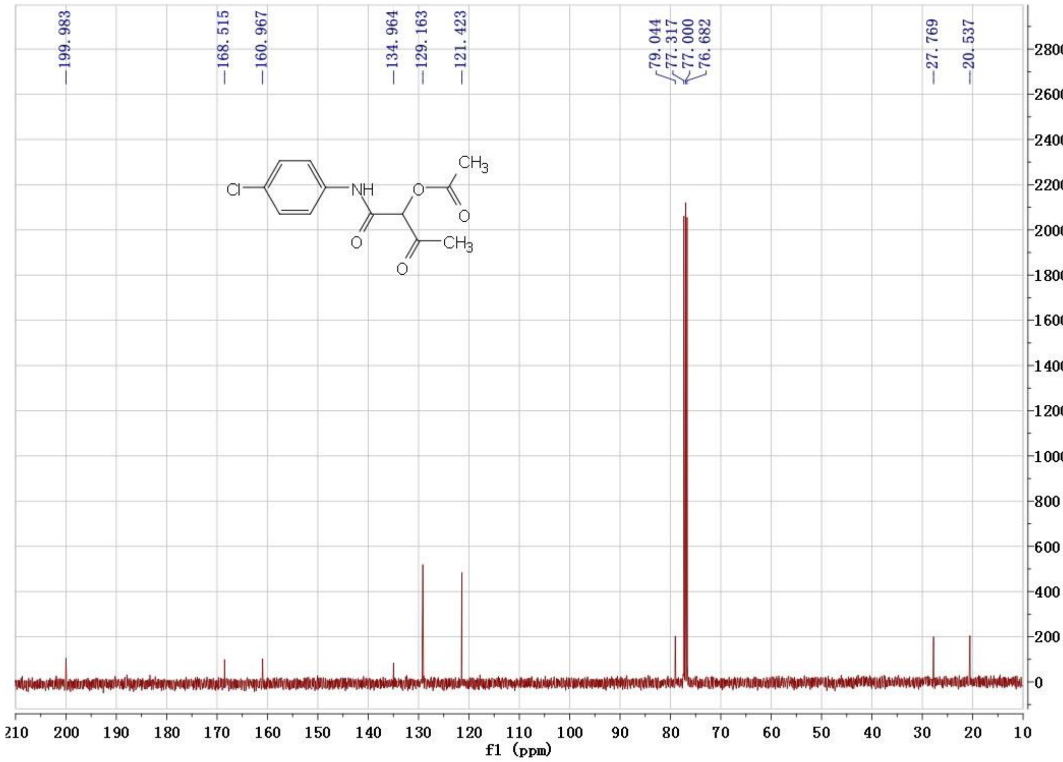
199.983
168.515
160.967
134.964
129.163
121.423
79.044
77.317
77.000
76.682
27.769
20.537
Cl
NH
O
CH3
f1 (ppm)

2e

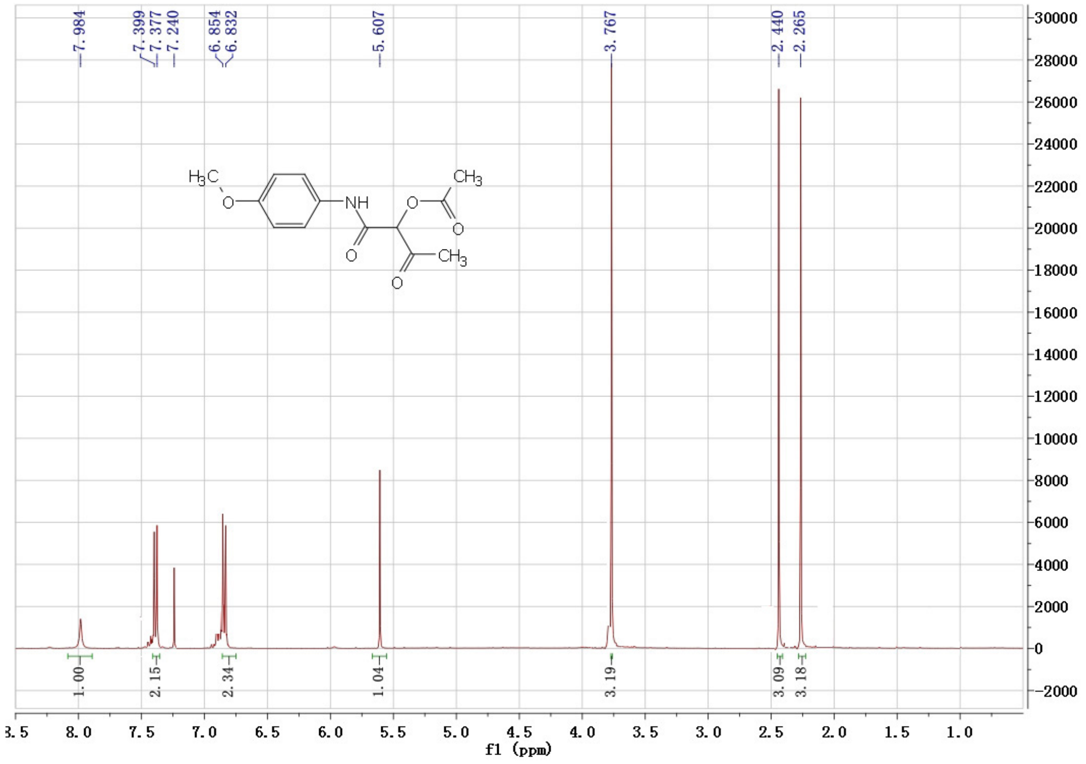

7.984
7.399
7.377
7.240
6.854
6.832
5.607
3.767
2.440
2.265
1.00
2.15
2.34
1.04
3.19
3.09
3.18
f1 (ppm)

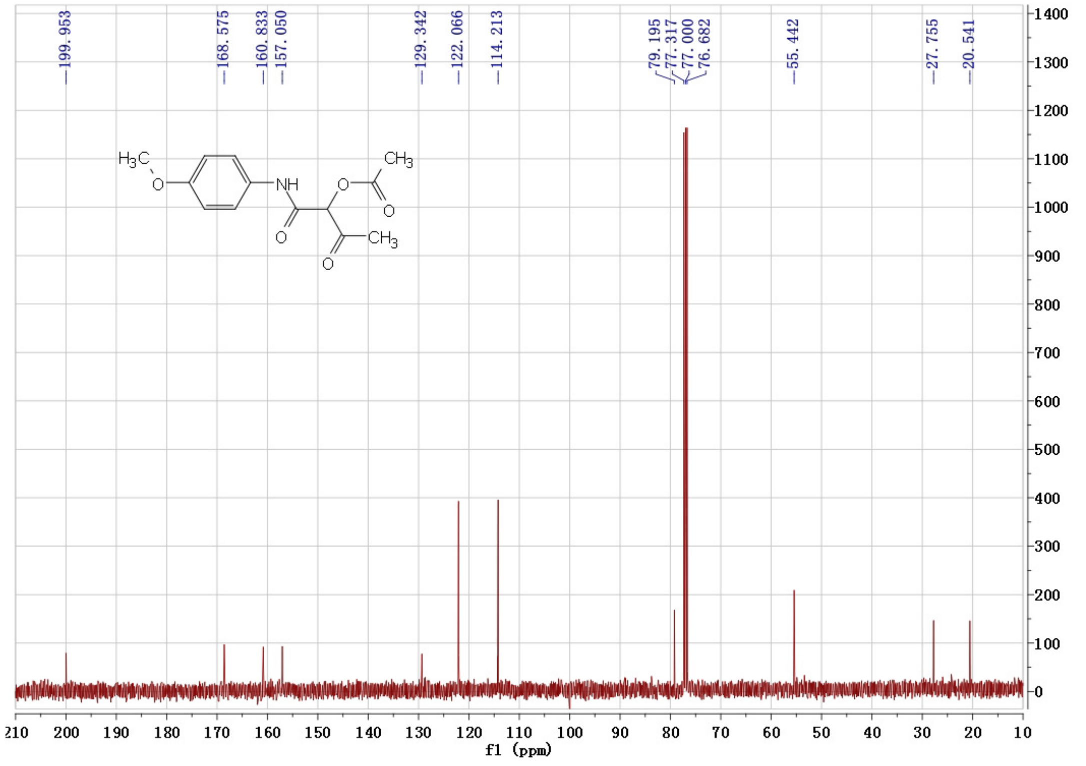

199.953
168.575
160.833
157.050
129.342
122.066
114.213
79.195
77.317
77.000
76.682
55.442
27.755
20.541
f1 (ppm)

2f

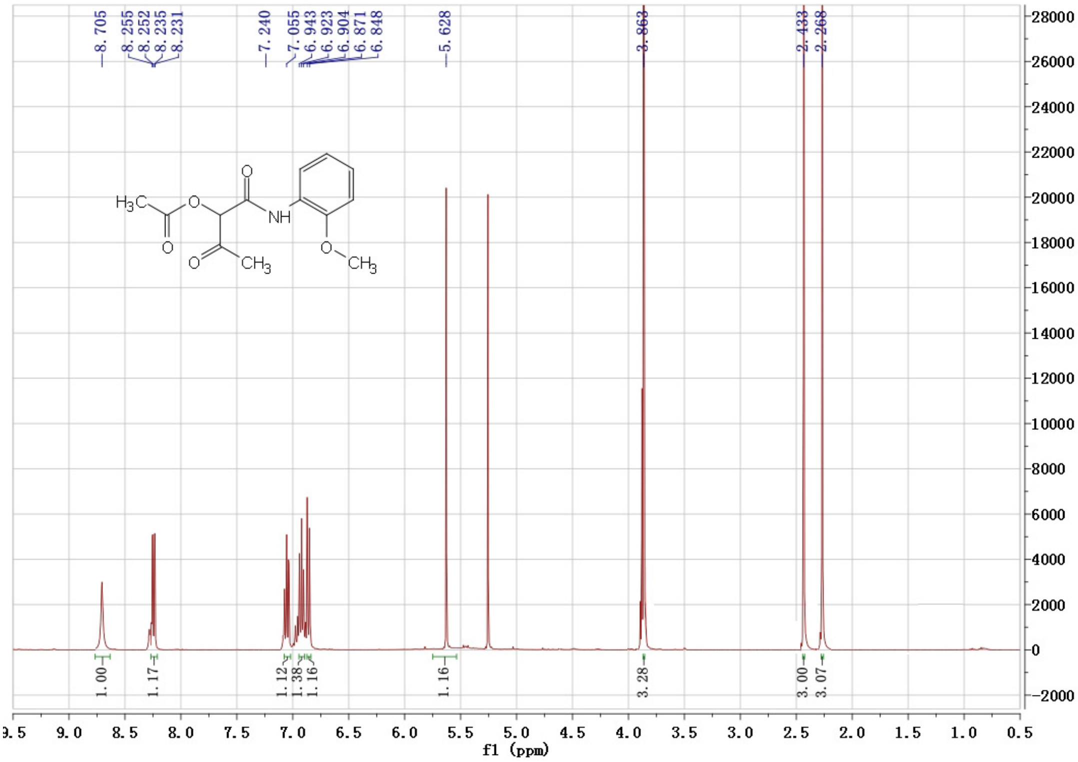

8.705
8.255
8.252
8.235
8.231
7.240
7.055
6.943
6.923
6.904
6.871
6.848
5.628
3.863
2.433
2.268
1.00
1.17
1.12
1.38
1.16
1.16
3.28
3.00
3.07
f1 (ppm)

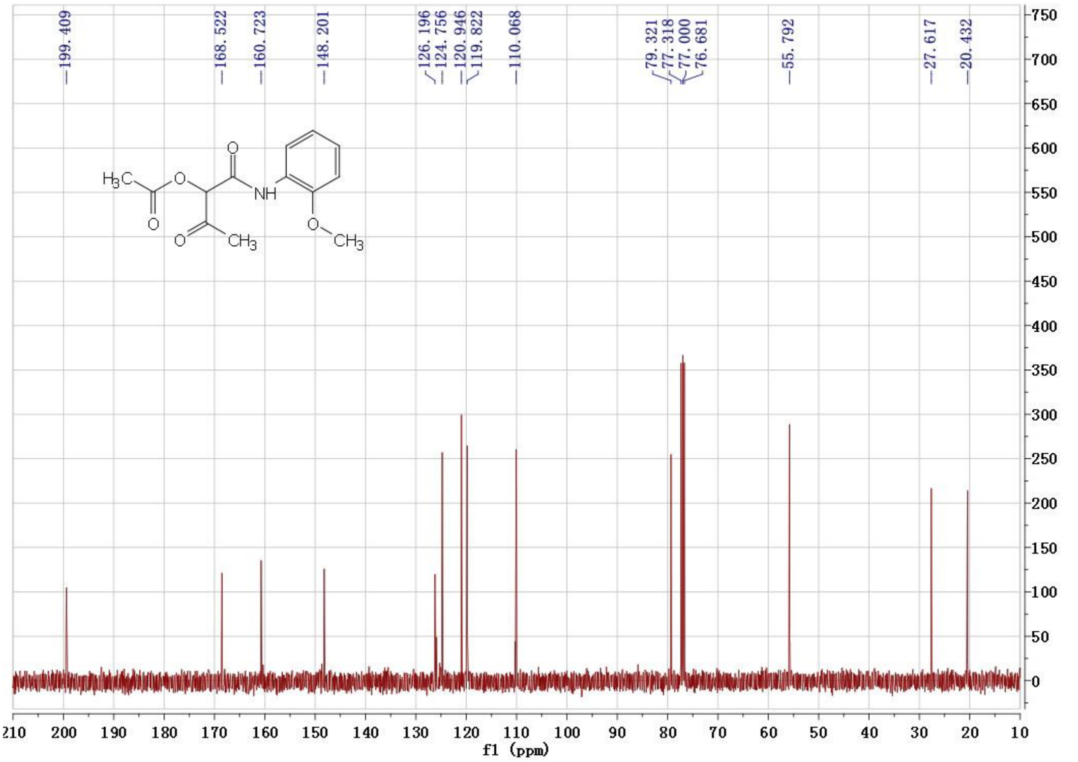

199.409
168.522
160.723
148.201
126.196
124.756
120.946
119.822
110.068
79.321
77.318
77.000
76.681
55.792
27.617
20.432
f1 (ppm)

2g

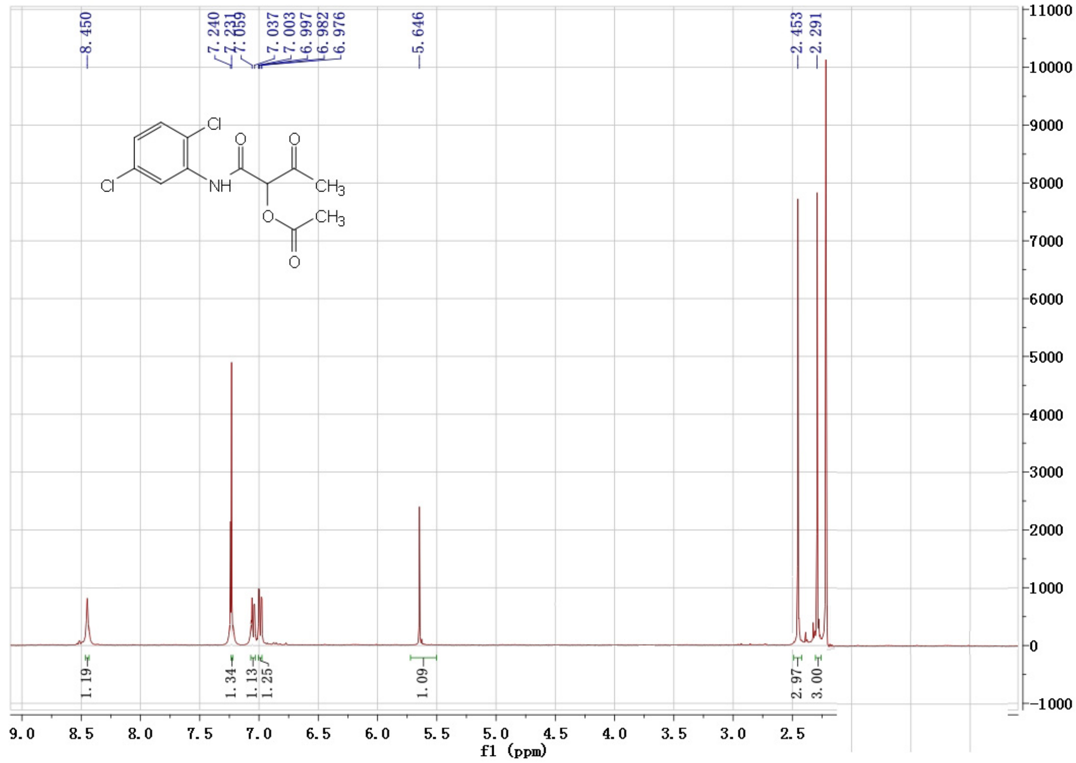

8.450
7.240
7.231
7.059
7.037
7.003
6.997
6.982
6.976
5.646
2.453
2.291
1.19
1.34
1.13
1.25
1.09
2.97
3.00
f1 (ppm)

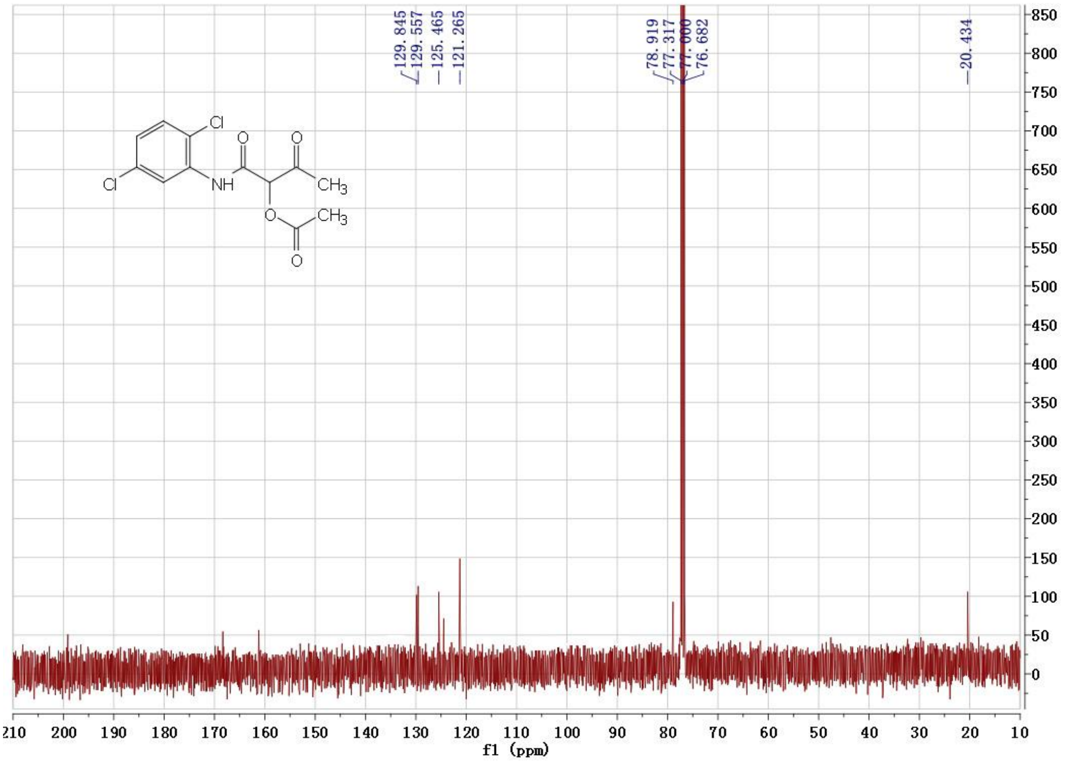

129.845
129.557
125.465
121.265
78.919
77.317
76.682
20.434
f1 (ppm)

2h

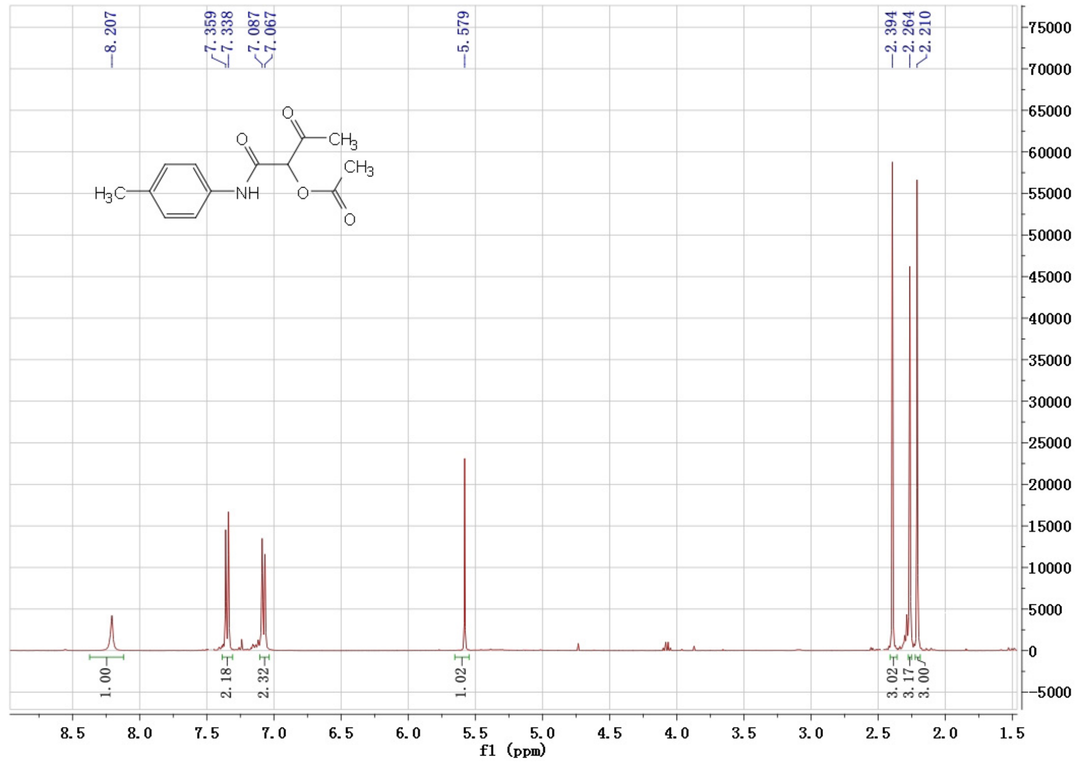
8.207
7.359
7.338
7.087
7.067
5.579
2.394
2.264
2.210
O
CH3
O
CH3
H3C
NH
O
O
1.00
2.18
2.32
1.02
3.02
3.17
3.00
75000
70000
65000
60000
55000
50000
45000
40000
35000
30000
25000
20000
15000
10000
5000
0
-5000
8.5
8.0
7.5
7.0
6.5
6.0
5.5
5.0
4.5
4.0
3.5
3.0
2.5
2.0
1.5
f1 (ppm)

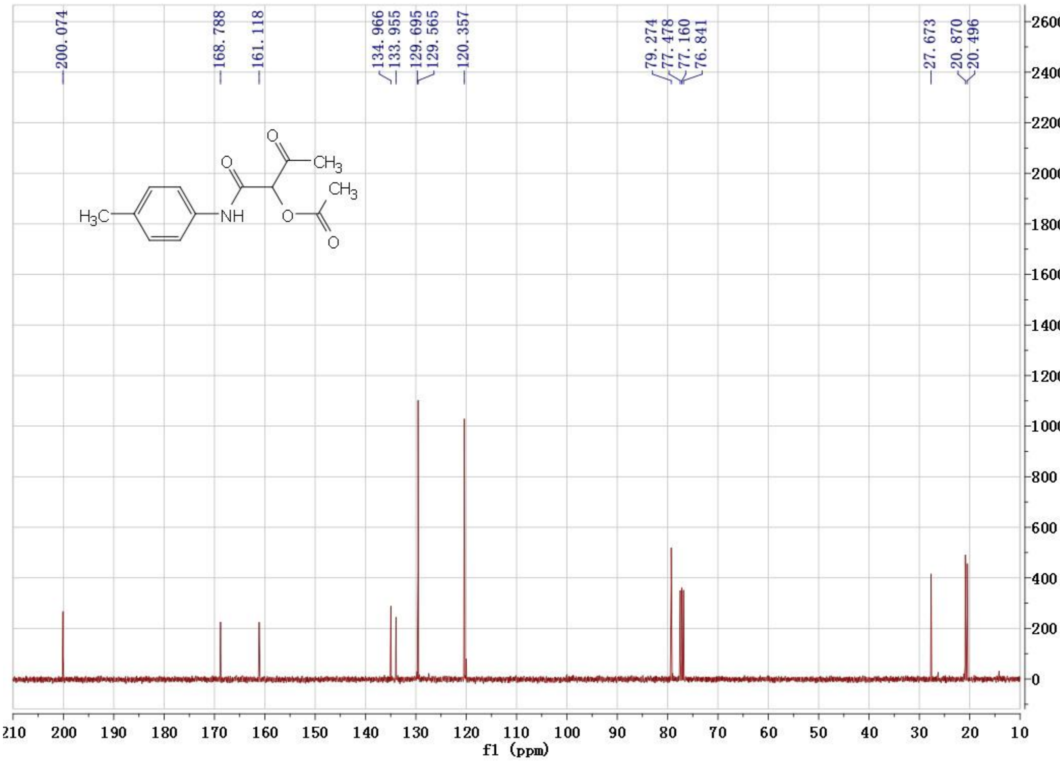
200.074
168.788
161.118
134.966
133.955
129.695
129.565
120.357
79.274
77.478
77.160
76.841
27.673
20.870
20.496
O
CH3
O
CH3
H3C
NH
O
O
2600
2400
2200
2000
1800
1600
1400
1200
1000
800
600
400
200
0
210
200
190
180
170
160
150
140
130
120
110
100
90
80
70
60
50
40
30
20
10
f1 (ppm)

2i

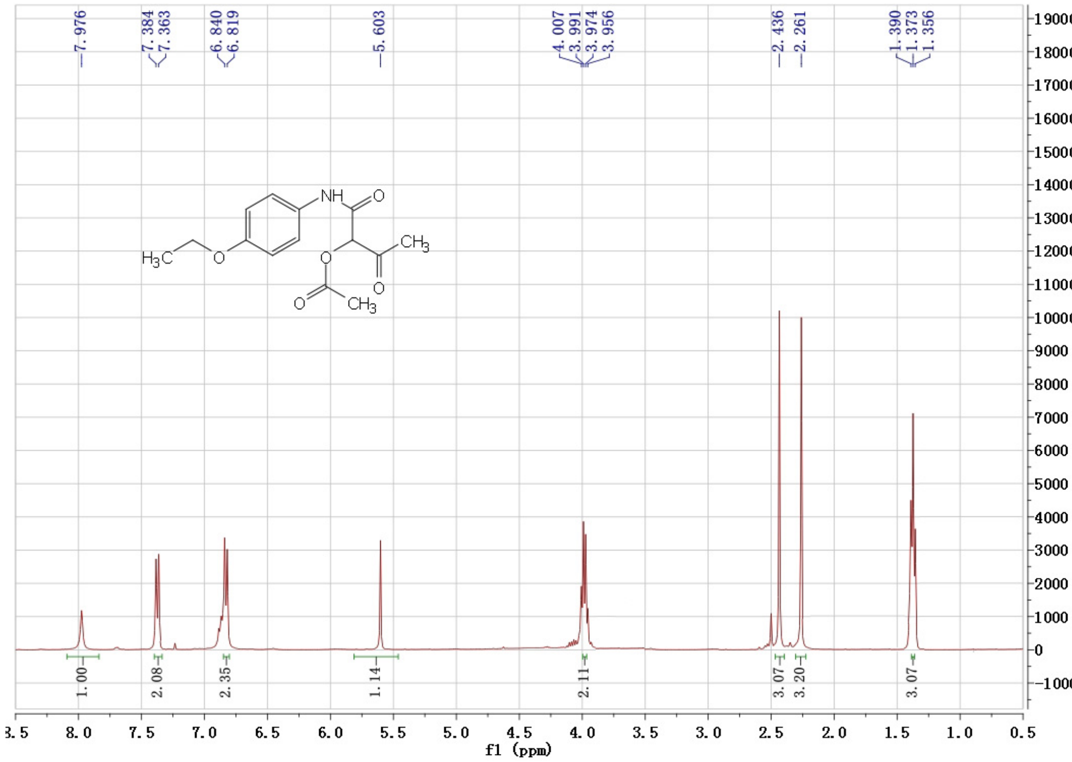

7.976
7.384
7.363
6.840
6.819
5.603
4.007
3.991
3.974
3.956
2.436
2.261
1.390
1.373
1.356
NH
O
H3C
O
O
CH3
O
O
CH3
1.00
2.08
2.35
1.14
2.11
3.07
3.20
3.07
f1 (ppm)

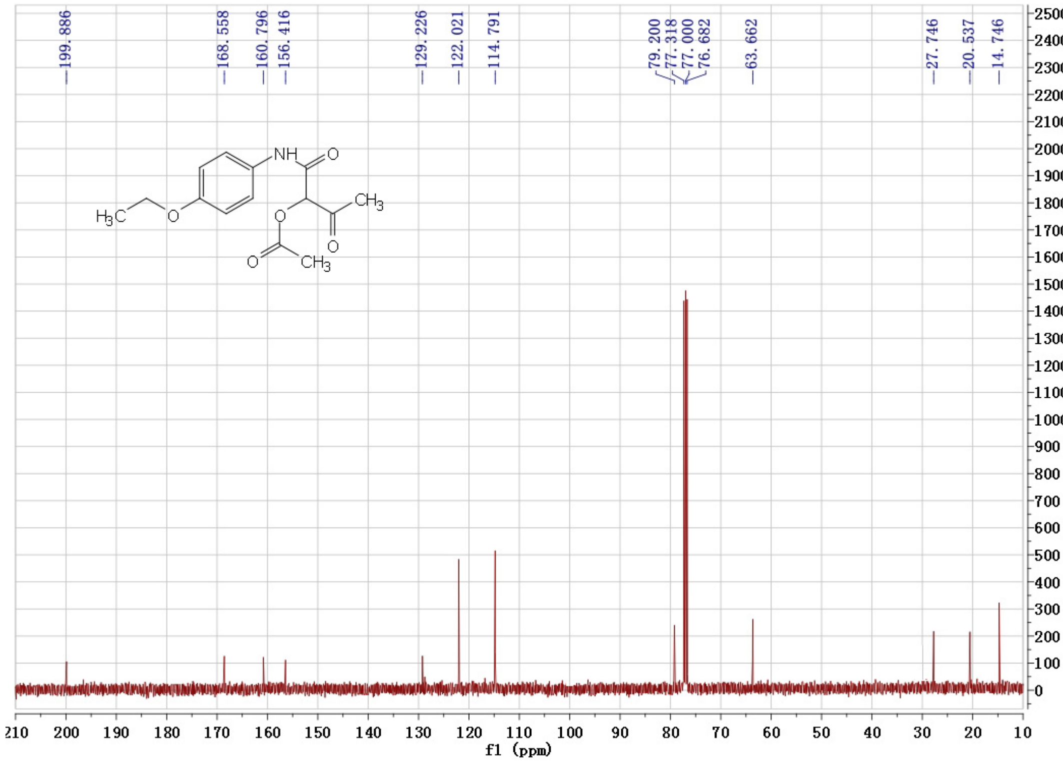

199.886
168.558
160.796
156.416
129.226
122.021
114.791
79.200
77.318
77.000
76.682
63.662
27.746
20.537
14.746
NH
O
H3C
O
O
CH3
O
O
CH3
f1 (ppm)

2j

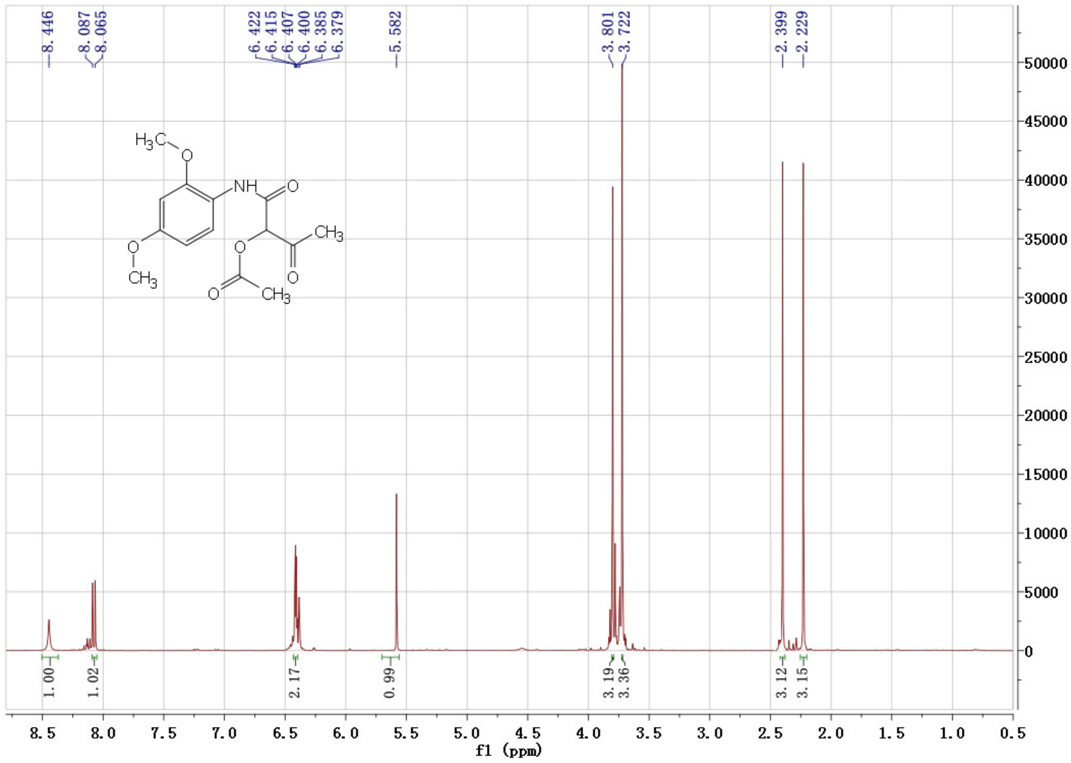

8.446
8.087
8.065
6.422
6.415
6.407
6.400
6.385
6.379
5.582
3.801
3.722
2.399
2.229
1.00
1.02
2.17
0.99
3.19
3.36
3.12
3.15
f1 (ppm)

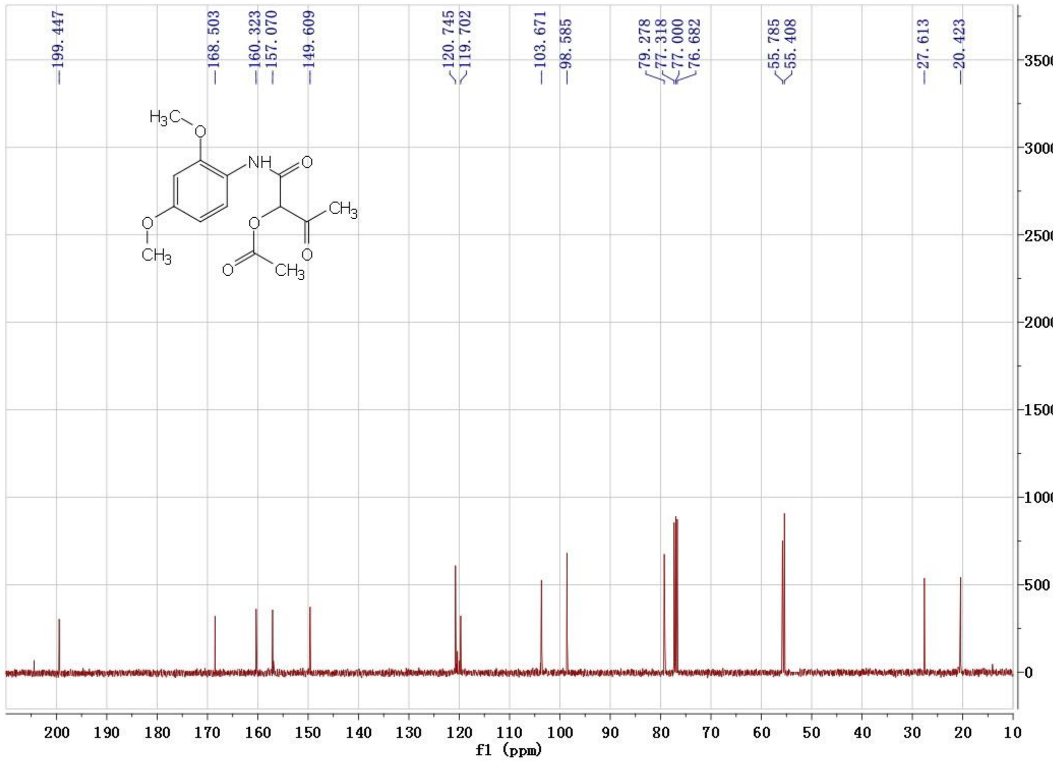

199.447
168.503
160.323
157.070
149.609
120.745
119.702
103.671
98.585
79.278
77.318
77.000
76.682
55.785
55.408
27.613
20.423
f1 (ppm)

2k

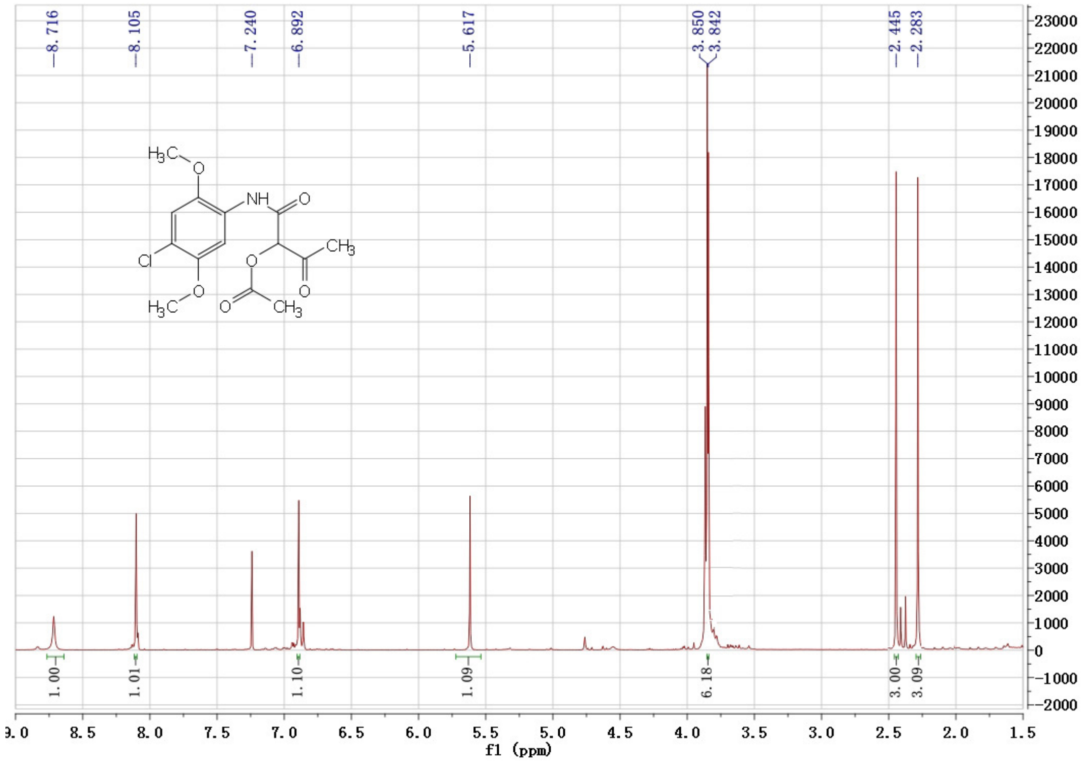
8.716
8.105
7.240
6.892
5.617
3.850
3.842
2.445
2.283
1.00
1.01
1.10
1.09
6.18
3.00
3.09
f1 (ppm)

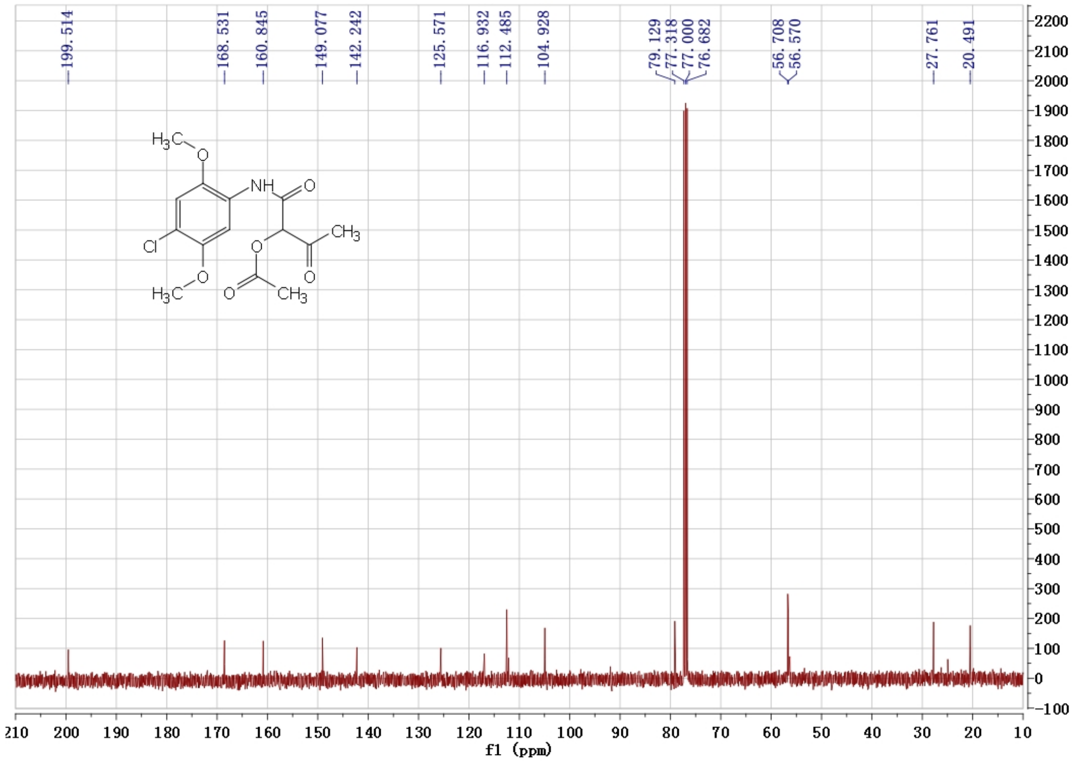
199.514
168.531
160.845
149.077
142.242
125.571
116.932
112.485
104.928
79.129
77.318
77.000
76.682
56.708
56.570
27.761
20.491
f1 (ppm)

2l

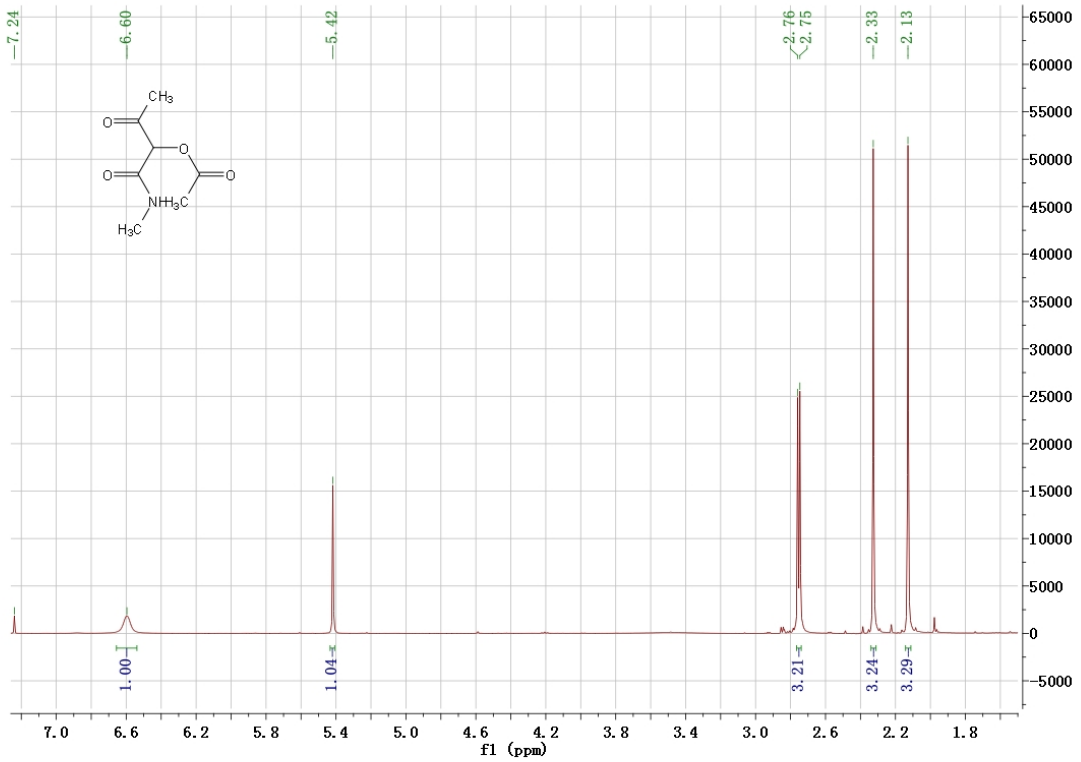

7.24
6.60
5.42
2.76
2.75
2.33
2.13
CH3
O
O
O
O
NH
H3C
H3C
1.00
1.04
3.21
3.24
3.29
7.0
6.6
6.2
5.8
5.4
5.0
4.6
4.2
3.8
3.4
3.0
2.6
2.2
1.8
f1 (ppm)
65000
60000
55000
50000
45000
40000
35000
30000
25000
20000
15000
10000
5000
0
-5000

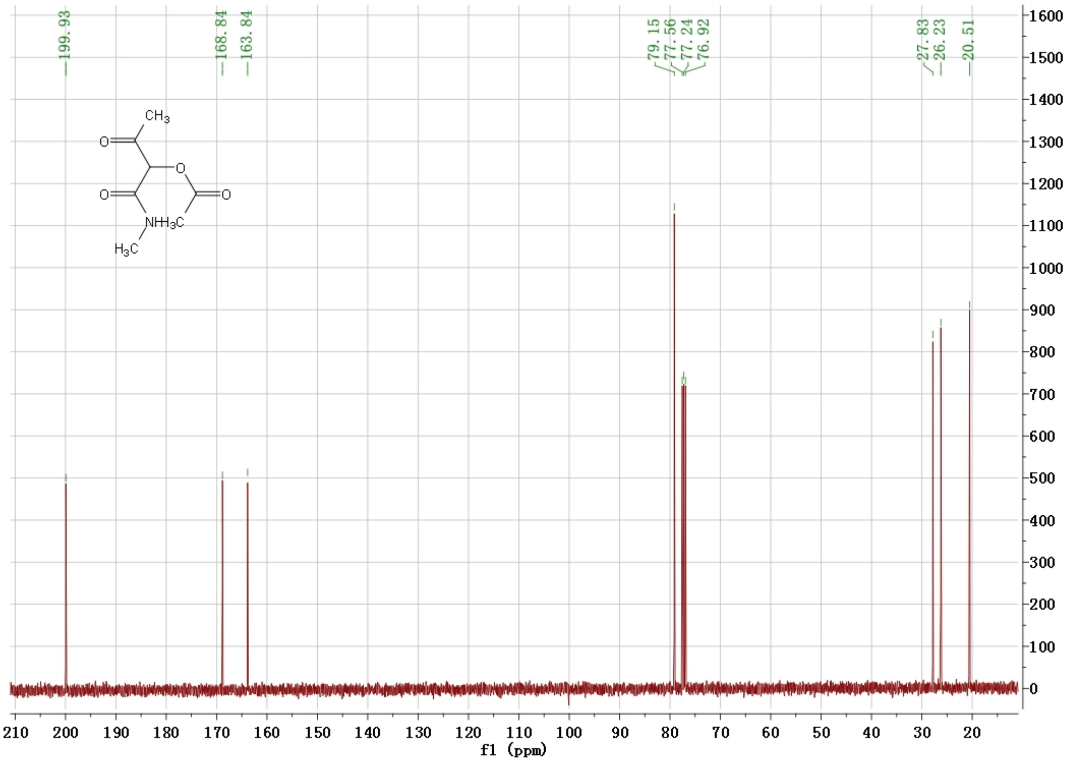

199.93
168.84
163.84
79.15
77.56
77.24
76.92
27.83
26.23
20.51
CH3
O
O
O
O
NH
H3C
H3C
210
200
190
180
170
160
150
140
130
120
110
100
90
80
70
60
50
40
30
20
f1 (ppm)
1600
1500
1400
1300
1200
1100
1000
900
800
700
600
500
400
300
200
100
0

2m

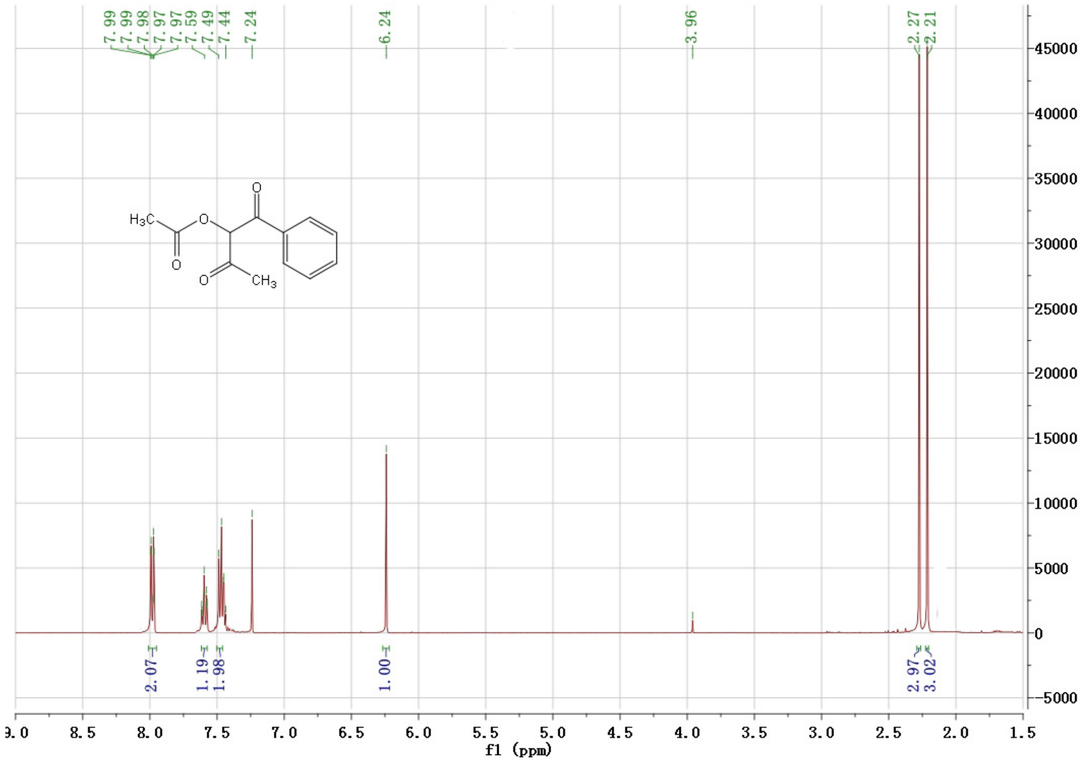
7.99
7.99
7.98
7.97
7.97
7.59
7.49
7.44
7.24
6.24
3.96
2.27
2.21
2.07
1.19
1.98
1.00
2.97
3.02
f1 (ppm)

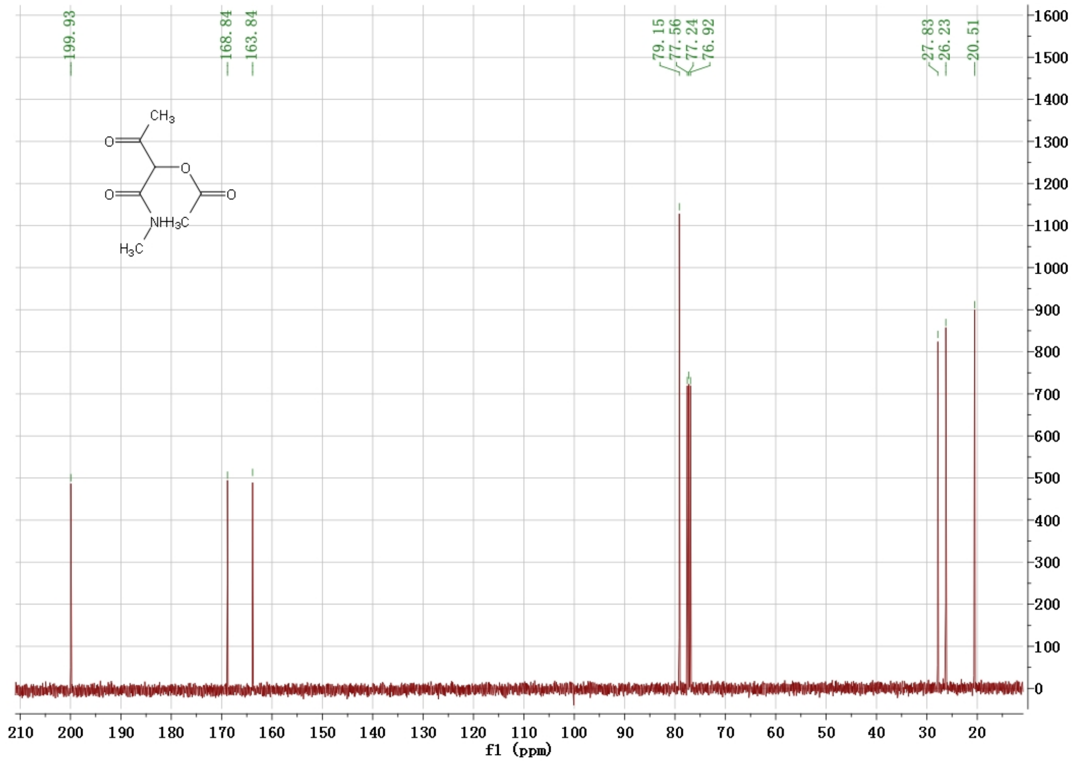
199.93
168.84
163.84
79.15
77.56
77.24
76.92
27.83
26.23
20.51
f1 (ppm)

2n

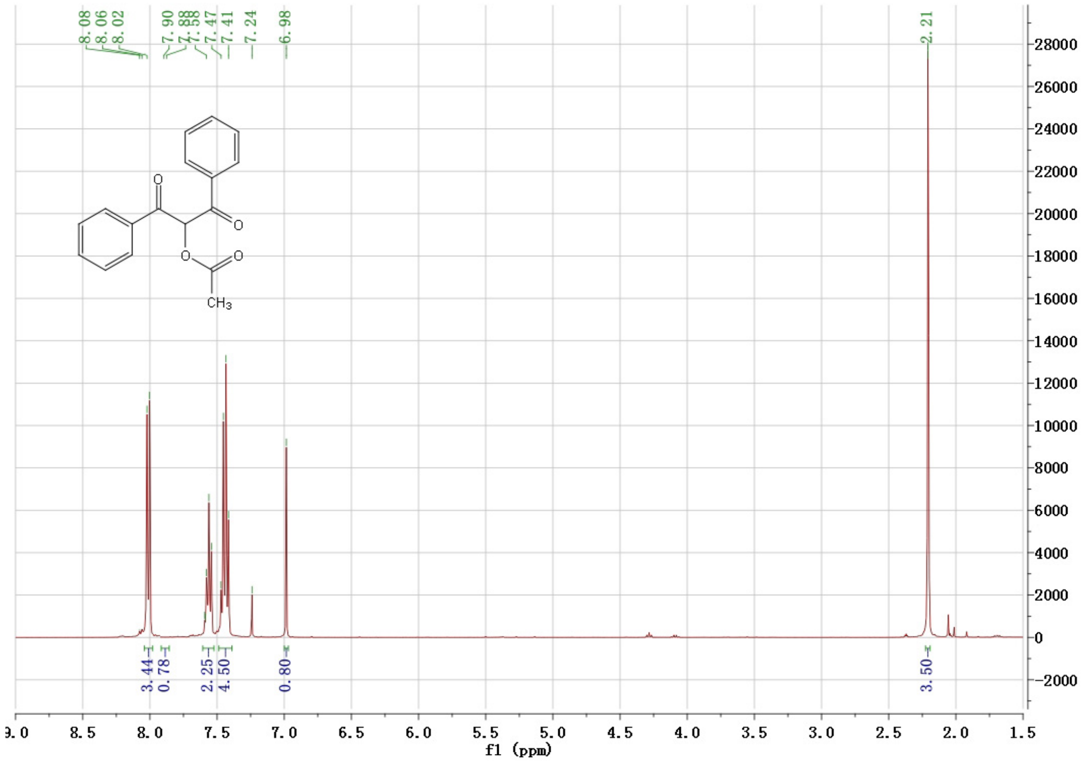
8.08
8.06
8.02
7.90
7.88
7.58
7.47
7.41
7.24
6.98
2.21
O
O
O
O
CH3
3.44
0.78
2.25
4.50
0.80
3.50
f1 (ppm)

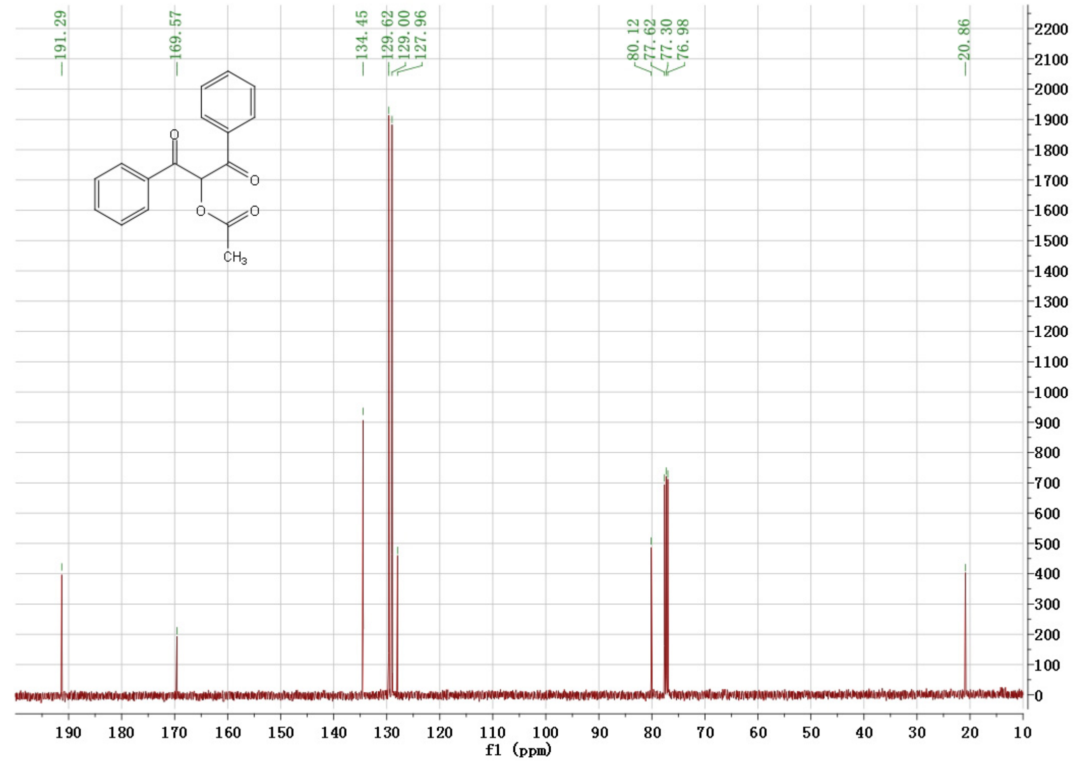
191.29
169.57
134.45
129.62
129.00
127.96
80.12
77.62
77.30
76.98
20.86
O
O
O
O
CH3
f1 (ppm)

2o

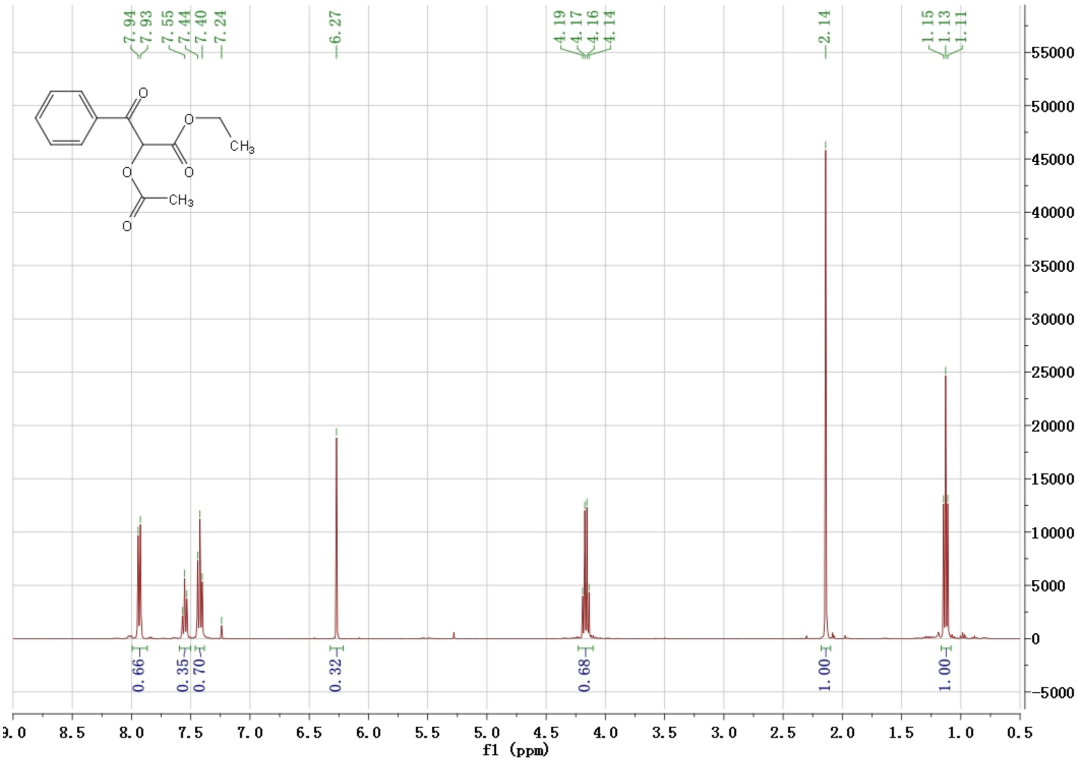
7.94
7.93
7.55
7.44
7.40
7.24
6.27
4.19
4.17
4.16
4.14
2.14
1.15
1.13
1.11
0.66
0.35
0.70
0.32
0.68
1.00
1.00
f1 (ppm)

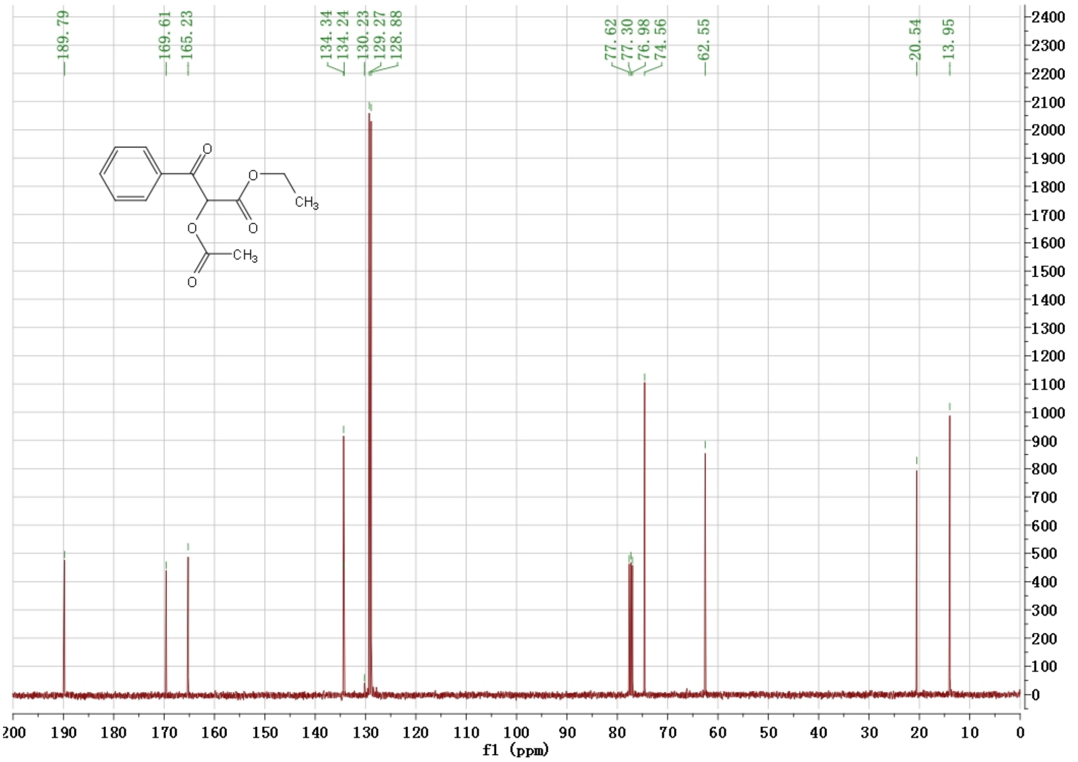
189.79
169.61
165.23
134.34
134.24
130.23
129.27
128.88
77.62
77.30
76.98
74.56
62.55
20.54
13.95
f1 (ppm)

References

[1] Han, M.; Nam, K. D.; Hahn, H. G.; Shin, D. Y. Tetrahedron Lett. 2008, 49, 5217–5219.

[2] Ahmed, Z.; Fischer, C.; Spannenberg, A.; Langer, P. Tetrahedron 2006, 62, 4800–4806.

[3] Yu, J.; Tian, J.; Zhanga, C. Adv. Synth. Catal. 2010, 352, 531–546.

[4] Altuna-Urquijo, M.; Gehre, A.; Stanforth , S. P.; Tarbit, P. Tetrahedron 2009, 65, 975–984.
